# SeTFiN: Self-Data Generation and Temporal-Frequency Fusion for Multivariate Sensor Time-Series Forecasting

**DOI:** 10.3390/s26196037

**Published:** 2026-09-23

**Authors:** Min-Seon Chae, Tae-Hyoung Park

**Affiliations:** Department of Intelligent Systems and Robotics, Chungbuk National University, Cheongju 28644, Republic of Korea; mschae@cbnu.ac.kr

**Keywords:** time-series forecasting, multivariate sensor data, self-data generation, temporal-frequency network, sensor data augmentation, Mamba

## Abstract

Multivariate sensor time-series forecasting is important for reliable decision-making in industrial environments. However, sensor observations can be affected by degradation patterns, including spikes, noise, shifts, stuck values, and missing observations, which may reduce forecasting accuracy. To address this problem, this paper proposes SeTFiN, a Self-Data Generation and Temporal-Frequency Fusion Network for multivariate sensor time-series forecasting. SeTFiN integrates degradation-oriented Self-Data Generation (SDG) with temporal-frequency fusion. During training, SDG applies domain-specific perturbations at corresponding temporal and frequency patch indices without requiring additional labeled degraded data. The architecture performs frequency-guided Cross-Domain Feature Rectification (CDFR) before expert encoding and adaptively fuses temporal and frequency features after expert modeling. Experiments were conducted on five public forecasting benchmarks and the real-world industrial Semiconductor Fabrication Dataset (SFD). SeTFiN achieved competitive performance on the public benchmarks. In the three-seed clean-SFD evaluation, it achieved the lowest overall average errors among the compared models, with an MSE of 11.628 and an MAE of 0.870. Under five controlled synthetic degradation conditions applied to the SFD test inputs, SDG reduced MSE across all degradation types, while its effect on MAE varied. SeTFiN also remained competitive in several long-lookback settings, although its relative performance depended on the dataset and input length. These results support the effectiveness of coordinated SDG and temporal-frequency fusion under the evaluated clean and controlled synthetic degradation conditions.

## 1. Introduction

Time-series sensor data are continuously generated in various industrial systems, including predictive maintenance, smart manufacturing, energy management, autonomous transportation, and industrial Internet of Things (IIoT) applications. These data reflect system states, operating conditions, and dynamic process changes over time [1,2]. With the increasing deployment of multiple sensors in cyber–physical systems (CPSs), multivariate time-series data have become an important information source for understanding complex industrial processes and supporting reliable decision-making [3]. As illustrated in Figure 1, multivariate sensor time-series forecasting aims to estimate future trajectories of multiple sensor variables from historical observations within a lookback window. This task is important because it supports proactive decision-making before system failures or performance degradation occur. However, forecasting industrial sensor data remains challenging because such data often contain long-range temporal dependencies, non-stationary dynamics, and complex inter-variable correlations [4].

A major challenge in reliable forecasting is sensor signal degradation. In real-world industrial environments, sensor observations may exhibit spikes, noise, shifts, stuck sensor values, and missing observations arising from communication failures, sensor aging, electrical interference, or unstable operating conditions [5,6,7,8]. Such degradation can alter the observed data distribution and reduce the accuracy of forecasting models trained primarily on clean observations. In this study, these degradation patterns are represented using five controlled synthetic degradation operators: spike, noise, shift, stuck value, and random masking. Random masking is used to simulate missing observations. Accordingly, robustness in this paper refers specifically to forecasting performance under the evaluated synthetic degradation conditions.

Time-series forecasting has been widely studied using temporal-domain models, including recurrent networks, Transformer-based models, convolutional models, and state-space models [2,9]. Representative long-term forecasting architectures such as Informer, Autoformer, TimesNet, and ModernTCN have improved forecasting performance on standard multivariate time-series forecasting benchmarks [10,11,12,13]. More recently, Mamba has shown strong potential for efficient long-sequence modeling with low computational complexity [14,15,16]. However, temporal-domain modeling alone may be insufficient for robust forecasting under industrial signal degradation. Transient fluctuations, localized oscillatory patterns, and subtle spectral changes caused by degradation can be difficult to distinguish directly in the time domain.

To complement temporal-domain modeling, recent studies have incorporated frequency-domain information into time-series forecasting [17,18,19,20]. Frequency-domain representations can reveal periodic components, localized spectral variations, and transient signal characteristics that are not clearly observed in the time domain [21]. In industrial sensor signals such as vibration, pressure, temperature, and current measurements, industrial signal degradation can induce subtle spectral changes that are difficult to capture using temporal-domain observations alone. Therefore, learning complementary temporal and frequency-domain representations is important for capturing both global temporal dependencies and local spectral patterns. However, existing frequency-domain and temporal-frequency forecasting studies have mainly focused on standard forecasting accuracy, and their static integration strategies may limit the learning of generalized and degradation-invariant representations under industrial signal degradation [22,23].

In addition to temporal-frequency representation learning, the choice of sequence modeling backbone is also important for robust forecasting. Transformer-based models are effective for modeling temporal dependencies, but their attention mechanism can increase computational cost as the input length grows. Mamba provides an alternative sequence modeling structure based on selective state spaces, which is suitable for long-range temporal dependency modeling with linear-time sequence processing [9,14,15,16]. Therefore, this study adopts Mamba as the temporal sequence modeling backbone and extends it within a temporal-frequency network for robust forecasting under industrial signal degradation.

To address these limitations, this paper proposes SeTFiN, a Self-Data Generation and Temporal-Frequency Fusion Network for multivariate sensor time-series forecasting under controlled synthetic sensor-degradation conditions. SeTFiN integrates degradation-oriented self-data generation with temporal-frequency representation learning without requiring additional degradation annotations. The self-data generation strategy introduces controlled synthetic perturbations that represent sensor degradation patterns, including spikes, noise, shifts, stuck sensor values, and random masking. The temporal-frequency architecture combines wavelet-based representations, CDFR, specialized temporal and frequency experts, and an adaptive fusion mechanism. The key novelty of SeTFiN lies in the coordinated interaction among degradation-oriented self-data generation, specialized temporal and frequency experts, and adaptive feature integration, enabling complementary temporal-frequency modeling and degradation-oriented representation learning within a unified forecasting framework. Experiments were conducted on five public multivariate time-series forecasting benchmarks and the real-world industrial SFD under clean and controlled synthetic degradation settings.

The main contributions of this paper are summarized as follows:

- We develop a degradation-oriented SDG strategy that applies domain-specific perturbations at corresponding patch indices in the temporal and frequency representations, preserving positional correspondence without requiring additional degradation annotations.
- We design a dual-stage temporal–frequency forecasting architecture that performs frequency-guided CDFR prior to expert sequence modeling and adaptively fuses representations from dedicated temporal and frequency Mamba experts.
- We conduct extensive evaluations on five public benchmarks and the real-world industrial SFD, demonstrating competitive forecasting accuracy and systematically assessing performance under multiple controlled synthetic sensor-degradation conditions and extended lookback settings.

## 2. Related Work

### 2.1. Multivariate Sensor Time-Series Forecasting

Multivariate sensor time-series forecasting has been studied for cyber–physical system (CPS) applications, including smart manufacturing, intelligent transportation, energy management, and industrial monitoring [2,3]. Early deep learning approaches, such as recurrent neural networks (RNNs) and long short-term memory (LSTM) networks, were widely used for sequential modeling, although recurrent processing can make long-range dependency modeling less efficient [2].

Transformer-based architectures have subsequently been applied to long-term time-series forecasting. Informer improves attention efficiency for long-sequence forecasting [10], while Autoformer introduces series decomposition and an auto-correlation mechanism [11]. FEDformer incorporates frequency-domain operations into a decomposition-based Transformer framework [17]. TimesNet and ModernTCN model temporal patterns using temporal 2D-variation and convolution-based architectures, respectively [12,13]. PatchTST segments time series into patches for channel-independent temporal modeling [24], whereas iTransformer treats individual variables as tokens to model inter-variable relationships [25]. DLinear provides a decomposition-based linear baseline for long-term forecasting [26].

State-space model (SSM)-based architectures have also been investigated for efficient long-sequence modeling. Structured state-space models provide a basis for scalable sequence modeling [9], and Mamba extends this direction through selective state-space updates [15]. Subsequent studies have adopted Mamba for forecasting: Wang et al. [14] investigated its empirical effectiveness, while TimeMachine [16] designed a multiMamba architecture for long sequences. More recent frequency-aware approaches include AdaMamba [22], which integrates context-dependent frequency analysis into state-space updates via adaptive frequency gating, and Cross-domain Time-Frequency Mamba [23], which couples separate temporal and frequency Mamba blocks with domain fusion. While these studies advance frequency-aware sequence modeling, systematic evaluation under the controlled sensor-degradation protocol considered here is not their primary focus.

### 2.2. Temporal-Frequency Representations for Forecasting

Time-series sensor signals can be analyzed in both temporal and frequency domains. Temporal representations describe sequential dynamics, whereas frequency-domain representations characterize periodic and spectral information. Wavelet analysis further provides localized multiscale time-frequency representations for non-stationary signals [21].

Forecasting methods have incorporated frequency-domain information through Fourier transforms, wavelet transforms, spectral decomposition, and frequency-aware learning. FEDformer uses Fourier- and wavelet-based frequency-enhanced blocks within a decomposition-based Transformer architecture [17]. Frequency-domain MLPs perform forecasting through spectral-domain learning [18], while FITS applies frequency interpolation for lightweight time-series forecasting [19]. Wavelet-based approaches use multiscale representations while retaining temporal localization [20,21].

Several recent studies integrate temporal and frequency-domain information within forecasting architectures. WaveRoRA applies multilevel discrete wavelet transform (DWT) and models multiscale wavelet coefficients using a mixing-splitting architecture and Rotary Route Attention [27]. AdaMamba incorporates adaptive frequency information directly into Mamba state-space updates [22], whereas Cross-domain Time-Frequency Mamba uses separate time- and frequency-domain Mamba components followed by domain fusion [23]. These approaches represent different strategies for integrating frequency information into time-series forecasting.

SeTFiN differs from these approaches by combining CWT-based frequency representation with dual-stage temporal-frequency interaction. Specifically, the front network performs frequency-guided temporal rectification, while the rear network uses separate temporal and frequency Mamba experts followed by adaptive fusion. In addition, SDG introduces representative degradation patterns into the temporal- and frequency-domain inputs during training. A schematic comparison with selected representative temporal-frequency forecasting approaches is provided in Appendix D Table A10.

### 2.3. Self-Data Generation for Degradation-Aware Forecasting

Forecasting performance may change when the characteristics of test observations differ from those encountered during training. In industrial environments, sensor measurements can be affected by noise, missing observations, and other signal degradation patterns [5,6,7]. In this study, five controlled synthetic degradation patterns are considered: spike, Gaussian noise, signal shift, stuck value, and random masking. These patterns serve as simplified proxies for abrupt outliers, measurement interference, calibration offsets, sensor freezing, and missing observations, respectively.

Perturbation-based and self-supervised methods have been investigated to increase training diversity and learn time-series representations. Temporal-frequency consistency-based contrastive learning uses temporal- and frequency-domain views for self-supervised representation learning [28], while masking-based methods reconstruct or learn from partially observed time-series inputs [29,30,31]. Generative approaches, including wavelet diffusion and diffusion autoregressive Transformers, have also been applied to time-series representation learning and forecasting [32,33]. Data-degradation strategies have additionally been considered in self-supervised time-series anomaly detection, as in AnomalyBERT [8], while other anomaly-detection approaches employ self-supervised, contrastive, or association-based learning objectives [34,35,36].

Within this broader context, SDG is formulated as a degradation-oriented data augmentation strategy for multivariate time-series forecasting. It generates perturbed training inputs by applying domain-specific operations to the temporal- and frequency-domain representations at corresponding patch positions. Accordingly, the contribution of SDG is evaluated under the corresponding controlled synthetic test-time degradation conditions.

### 2.4. Research Gap and Motivation

Multivariate sensor time-series forecasting has been extensively studied, but forecasting under degraded sensor observations has received comparatively less attention. Most existing forecasting methods are evaluated primarily under standard benchmark settings [10,11,12,13,14,24,25,26]. Their forecasting performance may change when the characteristics of the observed input signals differ from those encountered during training.

Temporal-frequency forecasting methods incorporate spectral and multiscale information into forecasting architectures [17,18,19,20,22,23,27], but their performance under representative sensor-degradation conditions has been less extensively evaluated. Similarly, existing perturbation and self-supervised learning approaches use masking, transformed views, or reconstruction-based objectives to learn time-series representations [28,29,30,31], but they are not specifically designed for multivariate forecasting under the controlled industrial sensor-degradation patterns considered in this study.

Motivated by these gaps, this paper proposes the Self-Data Generation and Temporal-Frequency Fusion Network (SeTFiN) for multivariate sensor time-series forecasting under controlled synthetic degradation conditions. SeTFiN combines SDG with temporal-frequency modeling by introducing controlled synthetic degradation patterns during training. The framework integrates CDFR, temporal and frequency Mamba experts, and adaptive rear fusion and is evaluated on standard benchmark datasets and under controlled synthetic sensor-degradation conditions.

## 3. Proposed Method

Figure 2 illustrates the overall architecture of SeTFiN. Given an input multivariate sensor time series $X\in R^{L\times C}$, where *L* denotes the lookback length and *C* denotes the number of sensor variables, SeTFiN forecasts future sensor values $\hat{Y}\in R^{H\times C}$ over the forecasting horizon *H*. The overall pipeline consists of temporal-frequency representation, self-data generation, and a forecasting network.

The temporal-frequency representation stage constructs complementary temporal and frequency-domain representations, where the frequency-domain representation is obtained using CWT. Self-data generation exposes the model to representative industrial signal degradation during training. The forecasting network processes the temporal and frequency-domain representations through the Front Network, Mamba temporal expert, Mamba frequency expert, Rear Network, and output module. Algorithm 1 summarizes the overall training procedure of SeTFiN.
**Algorithm 1:** Training Procedure of SeTFiN
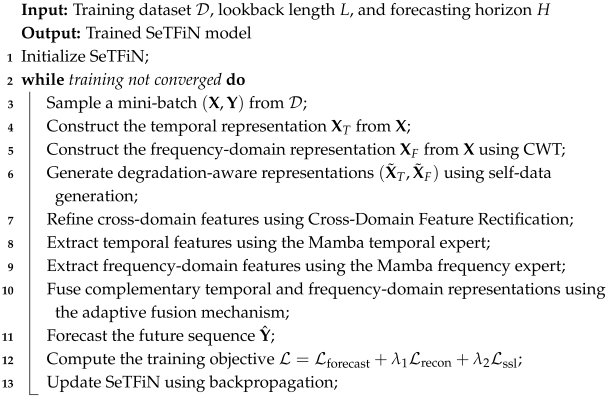


### 3.1. Preprocessing

Before feature encoding, SeTFiN performs preprocessing to construct input representations and expand the effective training distribution. Unlike conventional forecasting models that directly use original time-series observations, the preprocessing stage combines temporal-frequency representation construction with self-data generation.

The input time-series sensor data are first transformed into complementary temporal and frequency-domain representations to capture sequential dynamics and localized spectral characteristics. Self-data generation then simulates representative industrial signal degradation, including spikes, noise, shifts, stuck sensor values, and random masking, during training. This preprocessing stage provides degradation-aware inputs to the temporal-frequency network and supports the learning of generalized and degradation-invariant representations for robust forecasting.

#### 3.1.1. Temporal-Frequency Representation

SeTFiN constructs complementary temporal and frequency-domain representations from the input multivariate sensor time series. Given an input $X\in R^{L\times C}$, where *L* denotes the lookback length and *C* denotes the number of sensor variables, the temporal representation is defined as

(1) $$X_{T}=X.$$

To obtain the frequency-domain representation, SeTFiN applies the continuous wavelet transform (CWT) to the input sequence:

(2) $$X_{F}=W_{\mathrm{CWT}}\left(X\right),$$

where $W_{\mathrm{CWT}}\left(\cdot \right)$ denotes the CWT operation. CWT analyzes a signal using scaled and shifted wavelet functions and provides multiscale temporal and frequency localization. In this study, the Morlet wavelet is used as the mother wavelet for CWT to capture localized temporal and frequency variations in non-stationary sensor signals [21].

As illustrated in Figure 2, both $X_{T}$ and $X_{F}$ are partitioned into patch-level inputs. Temporal patches preserve local sequential patterns, while frequency-domain patches preserve localized spectral patterns. This patch partitioning retains local temporal and frequency-domain information before self-data generation is applied. After self-data generation, the degraded temporal and frequency-domain representations are denoted as ${\tilde{X}}_{T}$ and ${\tilde{X}}_{F}$, respectively. These representations are used as inputs to the subsequent temporal-frequency network.

Figure 3 shows an example of temporal and frequency-domain representations with patch partitioning constructed from the ETTh2 dataset. The frequency-domain representation reveals localized spectral patterns that are less apparent in the temporal-domain observation.

#### 3.1.2. Self-Data Generation

Forecasting performance may be affected when input signals exhibit degradation patterns that differ from those observed during training. To account for such variations, SDG is formulated as a degradation-oriented data augmentation procedure that generates perturbed training inputs using controlled synthetic sensor-degradation patterns.

Before applying SDG, SeTFiN first constructs the temporal- and frequency-domain representations from the same original input X, such that $X_{T}=X$ and $X_{F}=W_{\mathrm{CWT}}\left(X\right)$. Thus, the CWT is performed before the frequency-domain SDG operation and is not recomputed from the temporally perturbed input. The two representations are subsequently partitioned using corresponding patch indices.

As summarized in Algorithm 1, SDG is then applied to the patch-partitioned temporal- and frequency-domain representations during training. The perturbed representations are generated as

(3) $${\tilde{X}}_{T}=G_{T}\left(X_{T}\right),$$

(4) $${\tilde{X}}_{F}=G_{F}\left(X_{F}\right),$$

where $G_{T}\left(\cdot \right)$ and $G_{F}\left(\cdot \right)$ denote the SDG operations applied to the temporal and frequency representations, respectively.

In the temporal domain, SDG includes spike, Gaussian noise, signal shift, and stuck-value operators. One of these four operators is sampled for each temporal patch selected for degradation. Random masking is applied independently by replacing all values in the selected temporal patch with zeros. The temporal patch indices selected for degradation and masking are also used to identify the corresponding frequency-domain patch indices. At these indices, the frequency coefficients are attenuated and perturbed by additive Gaussian noise, while the coefficients at unselected indices remain unchanged. The sampling probabilities, perturbation parameters, and detailed formulations are provided in Appendix F Table A12.

Figure 4 illustrates the common patch-coordinate system and the position-preserving masking procedure. In this framework, temporal–frequency alignment is defined as patch-index correspondence rather than exact transform consistency. The temporal and frequency representations share the patch locations selected for perturbation, while distinct domain-specific operations are applied at these positions. This design avoids the additional computation required to recompute the CWT from the perturbed temporal sequences during training.

Figure 5 presents examples of the domain-specific perturbations applied to the temporal- and frequency-domain representations. At the representation level, the frequency-domain operation perturbs localized spectral features at the corresponding patch indices to reflect reduced spectral reliability across the degraded intervals, rather than simulating the exact CWT response of each temporal degradation operator. By introducing these controlled perturbations during training, SDG increases input diversity and exposes the model to multiple synthetic degradation patterns without requiring additionally collected degraded data or degradation annotations.

### 3.2. Forecasting Network

SeTFiN performs temporal–frequency interaction in two stages: frequency-guided temporal rectification in the Front Network and patch-wise adaptive fusion in the Rear Network. The forecasting network receives the temporal and frequency-domain representations, denoted by ${\tilde{X}}_{T}$ and ${\tilde{X}}_{F}$, from the preprocessing stage. During training, these representations may be either clean or synthetically perturbed depending on whether SDG is applied. As shown in Figure 2, the network consists of the Front Network, Mamba temporal expert, Mamba frequency expert, Rear Network, and output module. Algorithm 2 summarizes the forward procedure of the temporal–frequency network.
**Algorithm 2:** Forward Procedure of the Temporal-Frequency Network     **Input**:Temporal representation ${\tilde{X}}_{T}$ and degraded frequency-domain representation ${\tilde{X}}_{F}$     **Output**:Fused forecasting representation $F_{D}$Obtain rectified representations $X_{R}$ and $X_{RF}$ using the Front Network;Extract temporal features using the Mamba temporal expert: $F_{T}=M_{T}\left(X_{R}\right)$;Extract frequency-domain features using the Mamba frequency expert: $F_{F}=M_{F}\left(X_{RF}\right)$;Fuse $F_{T}$ and $F_{F}$ using the Rear Network with an adaptive fusion mechanism;Output the fused forecasting representation $F_{D}$;**return** $F_{D}$;

The Front Network first refines the input representations and produces the rectified representations $X_{R}$ and $X_{RF}$. The Mamba temporal expert extracts temporal features $F_{T}$ from $X_{R}$, while the Mamba frequency expert extracts frequency-domain features from $X_{RF}$ through convolutional and Mamba-based processing. The Rear Network then integrates the temporal and frequency-domain features using an adaptive fusion mechanism. Through this process, SeTFiN learns generalized and degradation-invariant representations for robust forecasting under industrial signal degradation.

#### 3.2.1. Front Network

Although preprocessing constructs complementary temporal and frequency-domain representations, the temporal representation may not explicitly reflect localized spectral variations. To address this issue, the Front Network performs Cross-Domain Feature Rectification (CDFR), as illustrated in Figure 6. CDFR uses frequency-domain information to refine the temporal representation before expert encoding.

Let ${\tilde{X}}_{T}$ and ${\tilde{X}}_{F}$ denote the degraded temporal and frequency-domain representations generated by self-data generation, respectively. Since ${\tilde{X}}_{F}$ contains scale-dependent frequency information, max pooling is first applied along the scale dimension:

(5) $$F_{\max}={\mathrm{MaxPool}}_{s}\left({\tilde{X}}_{F}\right),$$

where ${\mathrm{MaxPool}}_{s}\left(\cdot \right)$ denotes max pooling along the scale dimension. The resulting $F_{\max}$ is aligned with the temporal representation and used to generate a rectification gate.

The bottleneck MLP generates the gate feature as

(6) $$H_{F}=\mathrm{GELU}\left(W_{1}F_{\max}+b_{1}\right),\;Z_{gate}=W_{2}H_{F}+b_{2},$$

where $W_{1}$, $W_{2}$, $b_{1}$, and $b_{2}$ are learnable parameters. The rectification gate is then obtained by applying the sigmoid function:

(7) $$A=\sigma (Z_{gate}),$$

where σ(·) denotes the sigmoid function.

Finally, the rectified temporal representation is computed as

(8) $$X_{R}={\tilde{X}}_{T}+A⊙F_{\max},$$

where ⊙ denotes element-wise multiplication. The frequency-domain representation is preserved as $X_{RF}={\tilde{X}}_{F}$ for subsequent frequency-domain encoding. Through this process, CDFR selectively injects frequency-domain information into the temporal representation while preserving its sequential structure. The resulting $X_{R}$ and $X_{RF}$ are used as inputs to the Mamba temporal expert and Mamba frequency expert, respectively.

#### 3.2.2. Mamba Temporal Expert

After Cross-Domain Feature Rectification, the rectified temporal representation $X_{R}$ is forwarded to the Mamba-temporal expert. As illustrated in Figure 7, this module uses the Mamba block to extract temporal features from the refined temporal representation.

Mamba is based on a selective state space model (SSM), which enables linear-time sequence modeling for long input sequences [15]. Previous studies have also shown the applicability of Mamba-based architectures to time-series forecasting [14,16]. In SeTFiN, the Mamba-temporal expert adopts the original Mamba block without modifying its internal architecture. Given the rectified temporal representation $X_{R}$, the temporal feature representation is obtained as

(9) $$F_{T}=M_{T}\left(X_{R}\right),$$

where $M_{T}\left(\cdot \right)$ denotes the Mamba-temporal expert.

The extracted temporal feature $F_{T}$ captures sequential dependencies from $X_{R}$ and is delivered to the Rear Network. It is then integrated with the frequency-domain feature through the adaptive fusion mechanism.

#### 3.2.3. Mamba Frequency Expert

Complementary to the Mamba temporal expert, the Mamba frequency expert extracts frequency-domain features from the rectified frequency-domain representation $X_{RF}$. As shown in Figure 8, this expert consists of a 2D CNN block and a Mamba block. The 2D CNN block captures localized spectral patterns from $X_{RF}$, while the Mamba block encodes dependencies in the extracted frequency-domain features.

Given $X_{RF}$, the intermediate frequency-domain feature is defined as

(10) $$F_{C}=C\left(X_{RF}\right),$$

where C(·) denotes the convolutional feature extraction operator in the Mamba frequency expert. The resulting $F_{C}$ contains localized spectral information derived from the frequency-domain representation.

The intermediate feature is then encoded by the Mamba block as

(11) $$F_{F}=M_{F}\left(F_{C}\right),$$

where $M_{F}\left(\cdot \right)$ denotes the frequency encoding operator in the Mamba frequency expert. This design is consistent with recent studies that combine Mamba-based sequence modeling with frequency-aware representations for forecasting [15,22,23]. The resulting frequency-domain feature $F_{F}$ is forwarded to the Rear Network and integrated with the temporal feature $F_{T}$ through the adaptive fusion mechanism. Through this process, SeTFiN constructs fused temporal–frequency representations for forecasting under clean and controlled synthetic degradation conditions.

#### 3.2.4. Rear Network

The Rear Network generates the final forecasting representation by integrating the temporal feature $F_{T}$ and the frequency-domain feature $F_{F}$. As shown in Figure 9, the Rear Network uses an adaptive fusion mechanism because the relative contributions of temporal and frequency-domain information may vary across input sequences and degradation conditions.

Given $F_{T}$ and $F_{F}$ from the Mamba temporal expert and the Mamba frequency expert, respectively, the two features are first concatenated as

(12) $$X_{\mathrm{combined}}=F_{T}⊕F_{F},$$

where ⊕ denotes concatenation along the feature dimension.

The combined representation is then passed through a routing bottleneck:

(13) $$H_{\mathrm{route}}=\mathrm{GELU}\left(W_{r1}X_{\mathrm{combined}}+b_{r1}\right),$$

(14) $$Z_{\mathrm{route}}=W_{r2}H_{\mathrm{route}}+b_{r2},$$

where $W_{r1}$, $W_{r2}$, $b_{r1}$, and $b_{r2}$ are learnable parameters.

The routing weights are computed using the softmax function:

(15) $$\left[w_{\mathrm{time}},w_{\mathrm{freq}}\right]=\mathrm{Softmax}\left(Z_{\mathrm{route}}\right),\;w_{\mathrm{time}}+w_{\mathrm{freq}}=1.$$

The fused forecasting representation is obtained by weighted fusion:

(16) $$F_{D}=w_{\mathrm{time}}F_{T}+w_{\mathrm{freq}}F_{F}.$$

Based on $F_{D}$, the forecasting output is generated as

(17) $$\hat{Y}=P\left(F_{D}\right),$$

where P(·) denotes the forecasting projection head.

SeTFiN is optimized using the following training objective:

(18) $$L_{\mathrm{total}}=L_{\mathrm{forecast}}+\lambda_{1}L_{\mathrm{recon}}+\lambda_{2}L_{\mathrm{ssl}},$$

where $L_{\mathrm{forecast}}$ denotes the forecasting loss. The reconstruction loss is explicitly defined as

(19) $$L_{\mathrm{recon}}=\frac{1}{B}\sum_{i=1}^{B}\mathrm{MSE}\;\left({\hat{X}}_{\mathrm{recon}}^{\left(i\right)},X^{\left(i\right)}\right),$$

where ${\hat{X}}_{\mathrm{recon}}$ is the reconstructed input and *X* is the corresponding original clean input. The self-supervised loss follows the InfoNCE objective:

(20) $$L_{\mathrm{ssl}}=-\frac{1}{B}\sum_{i=1}^{B}\log\frac{\exp\;\left(\mathrm{sim}(z_{i},z_{i}^{+})/\tau \right)}{\sum_{j=1}^{B}\exp\;\left(\mathrm{sim}(z_{i},z_{j}^{+})/\tau \right)},$$

where $z_{i}$ is the normalized anchor representation and $z_{i}^{+}$ is obtained from the same sample using a lightly perturbed CWT view. Representations $z_{j}^{+}$ with j≠i correspond to other samples in the mini-batch and serve as negatives, and sim(·,·) denotes cosine similarity. The corresponding loss weights and SSL temperature are summarized in Table A13.

## 4. Experiments

### 4.1. Experimental Datasets

Experiments were conducted on six multivariate time-series datasets: ETTh1, ETTh2, ETTm1, ETTm2, Weather, and the Semiconductor Fabrication Dataset (SFD), as summarized in Table 1. The ETT datasets contain electricity transformer temperature measurements introduced for long-sequence time-series forecasting [10]. ETTh1 and ETTh2 are sampled hourly, whereas ETTm1 and ETTm2 are sampled at 15-min intervals. These datasets provide representative multivariate forecasting characteristics. The Weather dataset contains 21 meteorological variables and is widely used as a benchmark for long-term forecasting [11]. Its different variable dimensionality and temporal dynamics provide an additional heterogeneous forecasting setting.

SFD contains 17 gas-flow, pressure, and temperature variables collected from a semiconductor fabrication furnace [34]. Unlike the public forecasting benchmarks, SFD reflects a physically coupled, recipe-driven industrial process and provides a real-world manufacturing setting for evaluating SeTFiN. Detailed forecasting and degradation-aware evaluations on SFD are presented in Section 4.5.

### 4.2. Main Results

The standard forecasting performance of SeTFiN was evaluated on five public multivariate time-series benchmark datasets: ETTh1, ETTh2, ETTm1, ETTm2, and Weather. For each dataset, the original input sequence was retained as the temporal-domain representation, while the frequency-domain representation was obtained using the continuous wavelet transform (CWT) with the Morlet wavelet. The input sequence length was fixed to L=96, and the forecasting horizon was set to H∈{96, 192, 336, 720}, following commonly used long-term forecasting settings [10,11,12,17]. During training, SDG was applied to the temporal- and frequency-domain input patches using five degradation types: spike, Gaussian noise, shift, stuck value, and random masking.

SeTFiN was trained for up to 50 epochs using the AdamW optimizer with a learning rate of $1\times 10^{-4}$ and a weight decay of $1\times 10^{-4}$. Early stopping was based on the validation MSE. Experiments were conducted using random seeds 2023, 2024, and 2025. The resulting three-seed mean and sample standard deviation are reported in Appendix A Table A1, and the corresponding exploratory configuration-level comparisons are provided in Appendix A Table A2. MSE and MAE were used as the evaluation metrics, with lower values indicating better performance. All experiments were implemented in PyTorch (version 2.1.2) and conducted on a single NVIDIA H200 NVL GPU. The model and training hyperparameters of SeTFiN were fixed across datasets unless otherwise specified. Baseline models were evaluated under the same forecasting protocol using their official implementations and model-specific settings when available.

Table 2 presents the long-term forecasting results on the five public benchmark datasets. SeTFiN was compared with six baseline models: WaveRoRA, iTransformer, PatchTST, Informer, DLinear, and Autoformer. All results in Table 2 were obtained on the original clean test sets without additional test-time degradation. Therefore, Table 2 evaluates standard forecasting accuracy, while performance under degraded test inputs is evaluated separately in Section 4.5.4.

Across the five datasets and four forecasting horizons, SeTFiN achieved an overall average MSE of 0.351 and MAE of 0.374. Among the baseline models, iTransformer obtained the lowest overall average errors, with an MSE of 0.395 and MAE of 0.404. Relative to iTransformer, the average MSE and MAE of SeTFiN were lower by approximately 11.1% and 7.4%, respectively. SeTFiN achieved the lowest MSE in 15 of the 20 forecasting settings and the lowest MAE in 17 of the 20 settings.

At the dataset level, SeTFiN achieved the lowest average MSE on ETTh1, ETTh2, ETTm1, and ETTm2. On Weather, iTransformer achieved the lowest average MSE, whereas SeTFiN achieved the lowest MAE at all four forecasting horizons.

Compared with WaveRoRA, which also incorporates temporal-frequency information, SeTFiN recorded a lower overall average MSE (0.351 vs. 0.603) and MAE (0.374 vs. 0.534) under the same clean-test evaluation setting. A schematic comparison with selected representative temporal-frequency forecasting approaches is provided in Appendix D Table A10, summarizing differences in frequency representation, temporal-frequency interaction, sequence modeling, fusion strategy, and degradation-aware training.

We additionally evaluated the inference efficiency of SeTFiN against representative forecasting baselines. As shown in Appendix E Figure A1, SeTFiN requires longer inference time than some lightweight baselines, while its inference time remains relatively stable across the evaluated forecasting horizons on SFD.

Overall, Table 2 reports the standard forecasting performance of SeTFiN on clean benchmark test sets. Performance under controlled sensor degradation is evaluated separately on the industrial SFD dataset in Section 4.5.4.

### 4.3. Long-Lookback Forecasting Analysis

To evaluate forecasting performance with longer input sequences, we varied the lookback length as L∈{720, 1440, 2880, 5760} while fixing the forecasting horizon to H=96. In Table 3, SeTFiN was compared with WaveRoRA and iTransformer under the same long-lookback settings.

As shown in Table 3, among the models reported in the main table, SeTFiN achieved the lowest average MAE across all five benchmark datasets. For MSE, SeTFiN achieved the lowest average values on ETTh2, ETTm1, ETTm2, and Weather and tied with iTransformer for the lowest average MSE on ETTh1 (0.516). On ETTh2 and ETTm2, the average MSE of SeTFiN was 0.589 and 0.198, respectively, compared with 1.276 and 0.336 for iTransformer.

To provide an additional comparison with a recent Mamba-based forecasting model, TimeMachine was evaluated under the same forecasting settings. The three-seed results in Appendix B Table A3 show that TimeMachine achieved lower MSE than SeTFiN in 16 of the 20 public dataset–lookback configurations. In particular, the mean MSE of SeTFiN on ETTh2 increased from 0.325 at L=720 to 1.040 at L=5760, whereas TimeMachine achieved an MSE of 0.728 at L=5760. These results indicate that increasing the lookback length does not necessarily improve forecasting accuracy and that the relative performance of SeTFiN depends on the dataset and input length. Accordingly, SeTFiN remains competitive in several long-lookback settings, but its performance is not uniformly stable at extreme input lengths. Complete seed-level results are provided in Appendix B Table A5.

Figure 10 presents qualitative examples for L=1440 and H=96. In the selected examples, the forecasts produced by SeTFiN show smaller error regions than the compared baseline models on ETTh2, ETTm2, and Weather. These examples provide a qualitative comparison corresponding to the quantitative results in Table 3.

### 4.4. Ablation Study

To further validate the effectiveness of SeTFiN, we conducted ablation studies from two perspectives. First, we examined the effect of self-data generation under the L=H=96 forecasting setting. Second, we analyzed the contribution of the temporal and frequency-domain Mamba modules under the long-lookback setting.

#### 4.4.1. Effect of Self-Data Generation

To evaluate the contribution of self-data generation, we compared SeTFiN with and without SDG under the L=H=96 forecasting setting. As reported in Table 4, the model with SDG achieved lower MSE and MAE than the variant without SDG across all five benchmark datasets. On average, the MSE decreased from 0.282 to 0.273, and the MAE decreased from 0.330 to 0.321. These results show that SDG contributes to improved forecasting performance on the benchmark datasets.

#### 4.4.2. Effect of Temporal and Frequency-Domain Components

The contributions of the temporal- and frequency-domain components under extended-lookback conditions are examined separately through the component ablation study in Table 5.

To evaluate the contributions of the temporal and frequency-domain components under the final training condition, we conducted a component-wise ablation study with self-data generation enabled under the extended-lookback and short-horizon setting of L=720 and H=16. The temporal expert and the frequency-domain expert were selectively removed while the remaining architecture was kept unchanged. The w/o Temporal variant removes the temporal expert, whereas the w/o Frequency variant removes the frequency-domain expert. This setting evaluates the contribution of each component under the same SDG configuration used in the full SeTFiN model.

As shown in Table 5, SeTFiN (Ours) achieved the lowest average MSE and MAE across the five benchmark datasets. Compared with the w/o Temporal variant, the full model reduced the average MSE from 0.173 to 0.167 and the average MAE from 0.245 to 0.239. Compared with the w/o Frequency variant, the average MSE decreased from 0.170 to 0.167 and the average MAE from 0.242 to 0.239. These results show that using both temporal and frequency-domain components provides lower average forecasting errors than using either component alone under this setting.

The w/o Temporal variant showed slightly higher average MSE and MAE than the w/o Frequency variant, indicating a relatively larger contribution of the temporal component under the evaluated setting. However, removing the frequency-domain expert also increased both average MSE and MAE compared with the full model. Therefore, both components contribute to the forecasting performance of SeTFiN under the extended-lookback and short-horizon setting.

A comprehensive ablation study covering SDG, CDFR, adaptive fusion, the temporal and frequency experts, reconstruction loss, and self-supervised loss is provided for the industrial SFD in Appendix C Table A8. The results show that the temporal expert had the largest architectural contribution. Among the remaining components, removing SDG led to the largest increase in forecasting error under degradation conditions. In contrast, removing the self-supervised loss did not increase forecasting error under this setting, indicating that its contribution was setting-dependent.

### 4.5. Real-World Industrial Case Study: Semiconductor Fabrication Dataset

To evaluate SeTFiN in a real-world industrial manufacturing setting, we conducted an additional case study using SFD, which consists of measurements collected from a vertical low-pressure chemical vapor deposition (LPCVD) furnace. Unlike the public forecasting benchmarks, SFD contains physically coupled gas, pressure, and temperature measurements obtained from a recipe-driven semiconductor fabrication process. This case study evaluates SeTFiN in terms of forecasting accuracy, performance under controlled sensor-degradation conditions, and the contributions of selected model components.

#### 4.5.1. LPCVD System and Recipe-Driven Operations

The LPCVD system used for SFD collection is divided into a furnace process area and a loading area, as illustrated in Figure 11. The furnace process area consists of heater coils, a process tube, and profile thermocouples, whereas the loading area contains an auto shutter, a quartz boat, wafers, and a boat elevator.

The manufacturing recipe consists of eight sequential operational stages: wafer loading, boat elevation, vacuum evacuation, thermal ramp-up, thin-film deposition, chamber repressurization, boat lowering, and final purge and stabilization. Each stage changes the operating state of the furnace, and the corresponding gas flow, chamber pressure, and temperature measurements exhibit stage-dependent, nonstationary, and interdependent variations.

Figure 11 illustrates the eight operational stages, while Table 6 summarizes the corresponding recipe steps and their physical interpretations. For readability, only the stage indices and abbreviated stage names are shown in the figure.

#### 4.5.2. Sensor Variables and Experimental Protocol

SFD consists of 17 sensor variables organized into three categories: gas, pressure, and temperature, as summarized in Table 7. The nine gas variables measure the supply and circulation of precursor and purge gases used during the LPCVD process. The three pressure variables characterize the chamber vacuum and pressure conditions, whereas the five temperature variables measure thermal profiles across different furnace zones.

Measurements across these three sensor categories are coupled through the recipe-driven process. Changes in precursor or purge-gas flow can affect chamber pressure, and changes in the process state may also be associated with variations in furnace temperature profiles. Conversely, temperature variations can affect gas reactions and thin-film deposition behavior. SFD is therefore treated as a multivariate forecasting problem involving correlated sensor variables across gas, pressure, and temperature measurements.

The SFD observations were divided chronologically into 8416 training samples, 2104 validation samples, and 6312 test samples to preserve temporal order and prevent future information from being used during training. The industrial case study used a lookback length of L=720 and a forecasting horizon of H=16. Unless otherwise specified, all other preprocessing, model, and optimization settings were kept consistent with those described in Section 4.2.

#### 4.5.3. Forecasting Performance on the SFD

Table 8 presents the long-term forecasting performance of SeTFiN and six baseline models on the clean SFD test set. The lookback length was fixed to L=96, and the forecasting horizon was varied as H∈{96, 192, 336, 720}.

For the seed-2023 results reported in Table 8, SeTFiN achieved the lowest overall average MSE of 11.127 and MAE of 0.800 among the compared models. It obtained the lowest MSE across three of the four forecasting horizons (H∈{96, 192, 336}), while DLinear obtained the lowest MSE at H=720. For MAE, SeTFiN achieved the lowest value at all four forecasting horizons. In the corresponding three-seed comparison reported in Appendix A Table A1, SeTFiN achieved the lowest overall average errors, with an MSE of 11.628 and an MAE of 0.870.

Table 9 further evaluates forecasting performance under extended lookback lengths with the forecasting horizon fixed to H=96. The lookback length was varied as L∈{720, 1440, 2880, 5760}, and SeTFiN was compared with WaveRoRA and iTransformer. SeTFiN achieved the lowest MSE and MAE at all four lookback lengths. The average MSE and MAE of SeTFiN were 17.894 and 1.770, respectively, compared with 93.893 and 5.040 for WaveRoRA and 90.303 and 4.669 for iTransformer. These results show that SeTFiN maintained lower forecasting errors than the two compared models across the evaluated long-lookback settings.

#### 4.5.4. Performance Under Controlled Synthetic Sensor Degradation

To evaluate SeTFiN under controlled synthetic degradations of industrial sensor data, we considered five degradation conditions: spike, Gaussian noise, signal shift, stuck value, and random masking. These conditions were used to represent abrupt outliers, measurement noise, calibration offsets, sensor freezing, and missing sensor observations, respectively. The degradation operators were applied only to the test inputs, while the forecasting targets remained unchanged. Therefore, this evaluation examines forecasting performance under controlled synthetic corruptions rather than naturally occurring industrial sensor failures.

We compared SeTFiN trained with SDG with an otherwise identical variant trained without SDG. Both variants were evaluated under the same setting of L=720 and H=16. The results in Table 10 are reported using random seed 2023. The corresponding mean and sample standard deviation over three independent runs using random seeds 2023, 2024, and 2025 are provided in Appendix C Table A7.

For the results of seed 2023 shown in Table 10, SDG reduced the MSE under all five controlled synthetic test-time degradation conditions. The MSE decreased from 2.023 to 1.973 under spike, from 1.840 to 1.790 under Gaussian noise, from 1.801 to 1.710 under signal shift, from 1.864 to 1.762 under stuck value, and from 12.668 to 12.528 under random masking. The largest relative MSE reductions were observed under stuck value and signal shift, with reductions of approximately 5.47% and 5.05%, respectively.

The MAE results showed a different trend. Under random masking, SDG reduced the MAE from 1.270 to 1.201, corresponding to a reduction of approximately 5.43%. In contrast, the variant without SDG obtained lower MAE values under spike, Gaussian noise, signal shift, and stuck value. These results indicate that the effect of SDG depends on the degradation condition and evaluation metric. Because MSE penalizes large prediction errors more heavily through squared deviations, the consistent MSE reductions indicate reduced sensitivity to larger forecasting errors under degraded inputs, even though the MAE results are mixed. The three-seed results in Appendix C Table A7 also show lower mean MSE under all five conditions, while the effect on MAE varies across degradation types.

The five degradation operators provide simplified proxies for common sensor-quality problems: abrupt outliers, measurement interference, calibration offsets, sensor freezing, and missing observations. However, they do not reproduce the full severity, duration, temporal dependence, or operating-context dependence of naturally occurring industrial failures. Accordingly, the SFD results characterize forecasting performance under the evaluated clean and controlled synthetic degradation conditions and should not be interpreted as evidence of robustness to all real industrial sensor failures.

## 5. Conclusions

This paper proposed SeTFiN, a Self-Data Generation and Temporal-Frequency Fusion Network for multivariate sensor time-series forecasting under controlled synthetic sensor-degradation conditions. SeTFiN combines patch-coordinate-aligned, domain-specific perturbations with CDFR, temporal and frequency Mamba experts, and adaptive fusion. In the seed-2023 public-benchmark comparison, SeTFiN achieved an overall average MSE of 0.351 and MAE of 0.374, compared with 0.395 and 0.404 for iTransformer. On the clean industrial SFD, SeTFiN achieved the lowest overall average errors among the models included in the three-seed comparison, with an MSE of 11.628 and an MAE of 0.870.

In the three-seed long-lookback evaluation, TimeMachine achieved lower mean MSE in 16 of the 20 public dataset–lookback configurations, indicating that the relative performance of SeTFiN depends on the dataset and input length. Under five controlled synthetic degradation conditions applied to the SFD test inputs, SDG reduced mean MSE in all conditions, whereas its effect on MAE varied across degradation types. These results characterize performance under the evaluated clean and controlled synthetic conditions and do not establish robustness to naturally occurring industrial sensor failures.

The ablation results showed higher mean forecasting errors when CDFR, the temporal or frequency expert, adaptive fusion, or the reconstruction objective was removed. The temporal expert had the largest architectural impact. However, removing the self-supervised loss did not increase the forecasting error in the SFD ablation setting; therefore, the improvement observed in this setting cannot be attributed to the self-supervised objective.

Future work will evaluate SeTFiN across multiple degradation severity levels and more diverse degradation patterns, including variable-duration and context-dependent corruptions. It will also examine naturally occurring industrial sensor failures when suitable data become available and investigate methods for reducing the computational cost of the temporal-frequency network.   

## Figures and Tables

**Figure 1:**
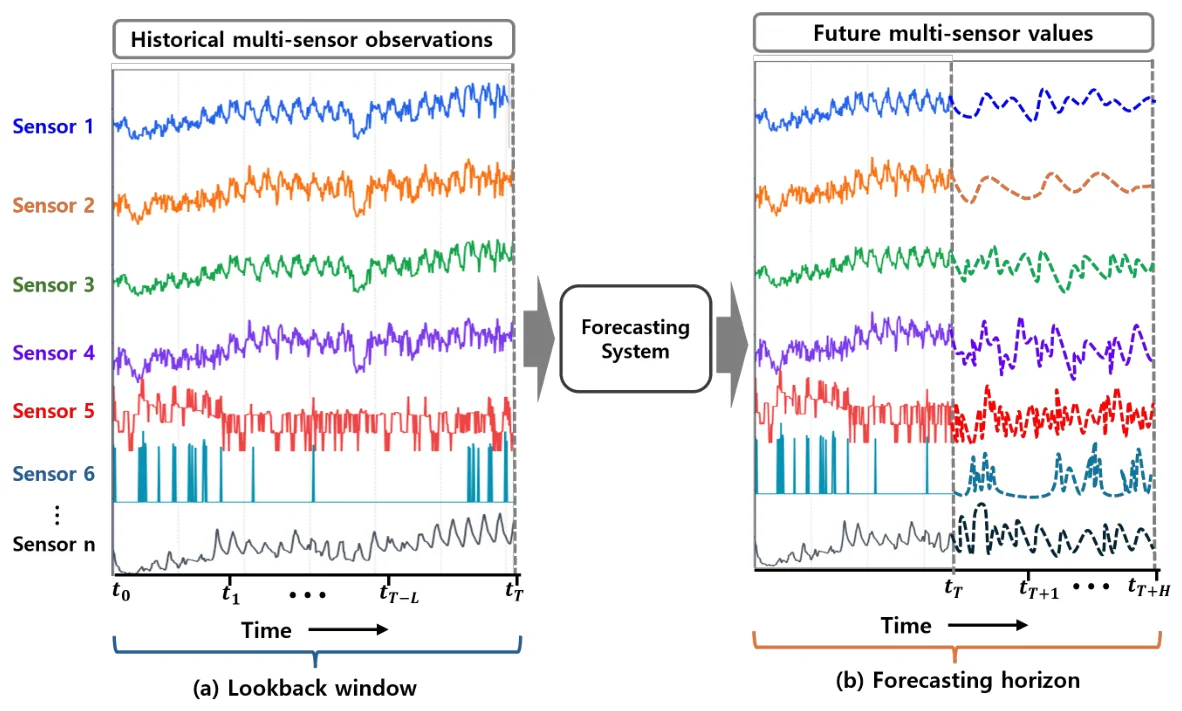
Conceptual illustration of multivariate sensor time-series forecasting. Given a lookback window containing historical observations from multiple sensors, the forecasting system estimates future multisensor values over a predefined forecasting horizon.

**Figure 2:**
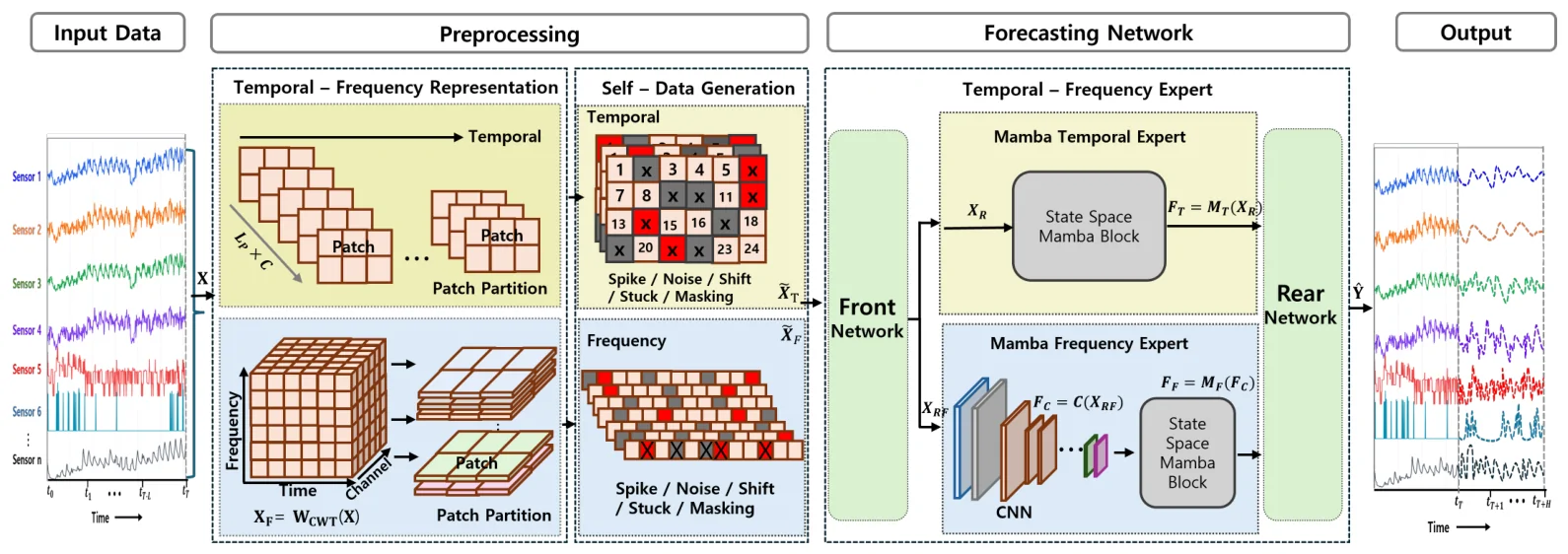
Overall architecture of the proposed SeTFiN framework. SeTFiN integrates self-data generation with temporal-frequency fusion for multivariate sensor time-series forecasting under industrial signal degradation.

**Figure 3:**
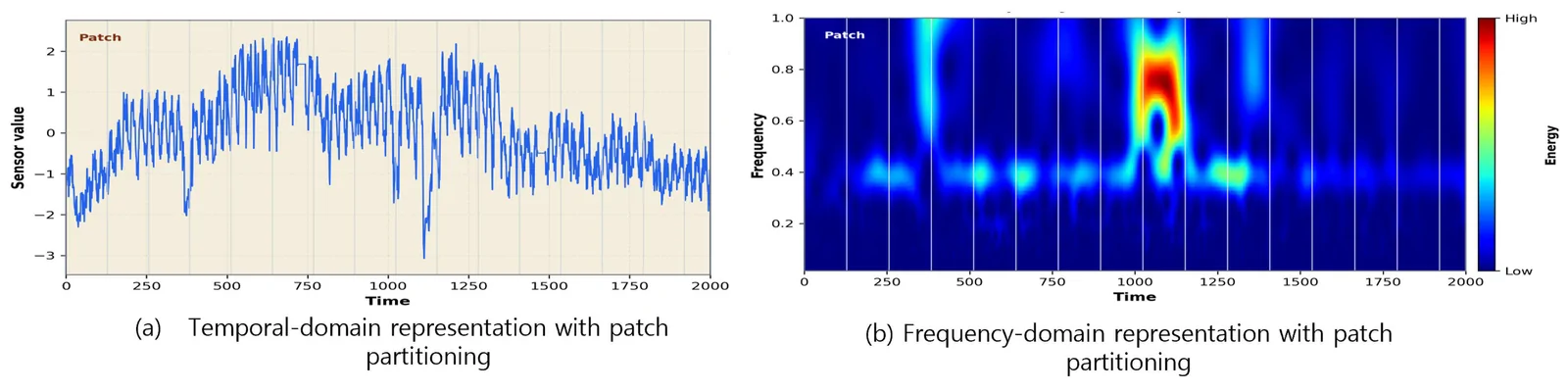
Example of temporal and frequency-domain representations with patch partitioning on the ETTh2 dataset.

**Figure 4:**
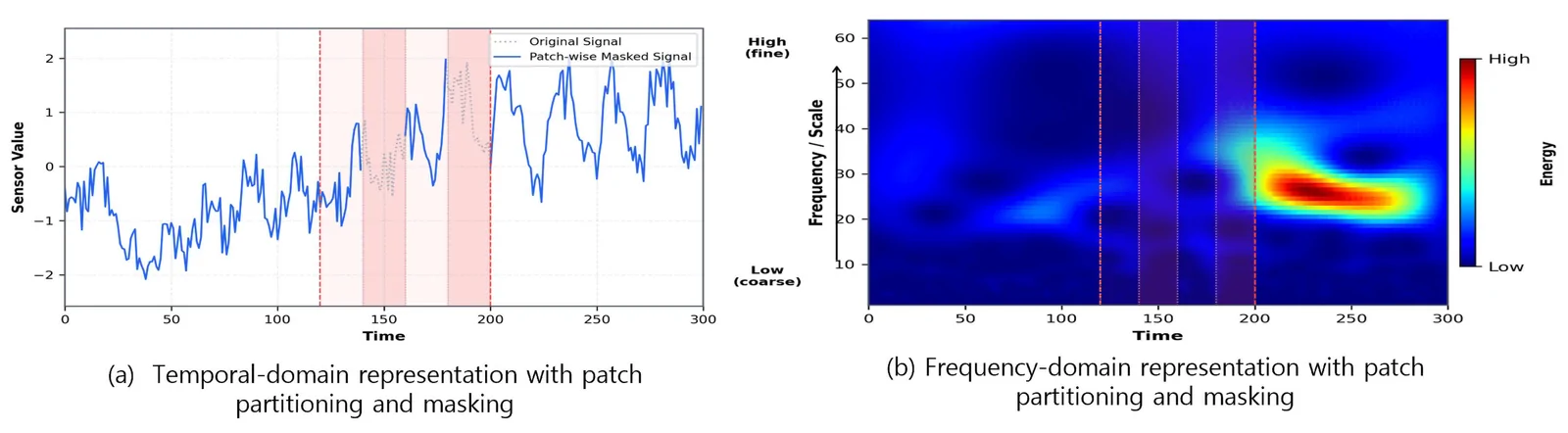
Illustration of the shared patch-coordinate system and patch-index correspondence used in SDG. (**a**) Temporal patches selected for degradation and independent random masking. (**b**) Corresponding frequency-domain patch indices selected for coefficient attenuation and Gaussian perturbation.

**Figure 5:**
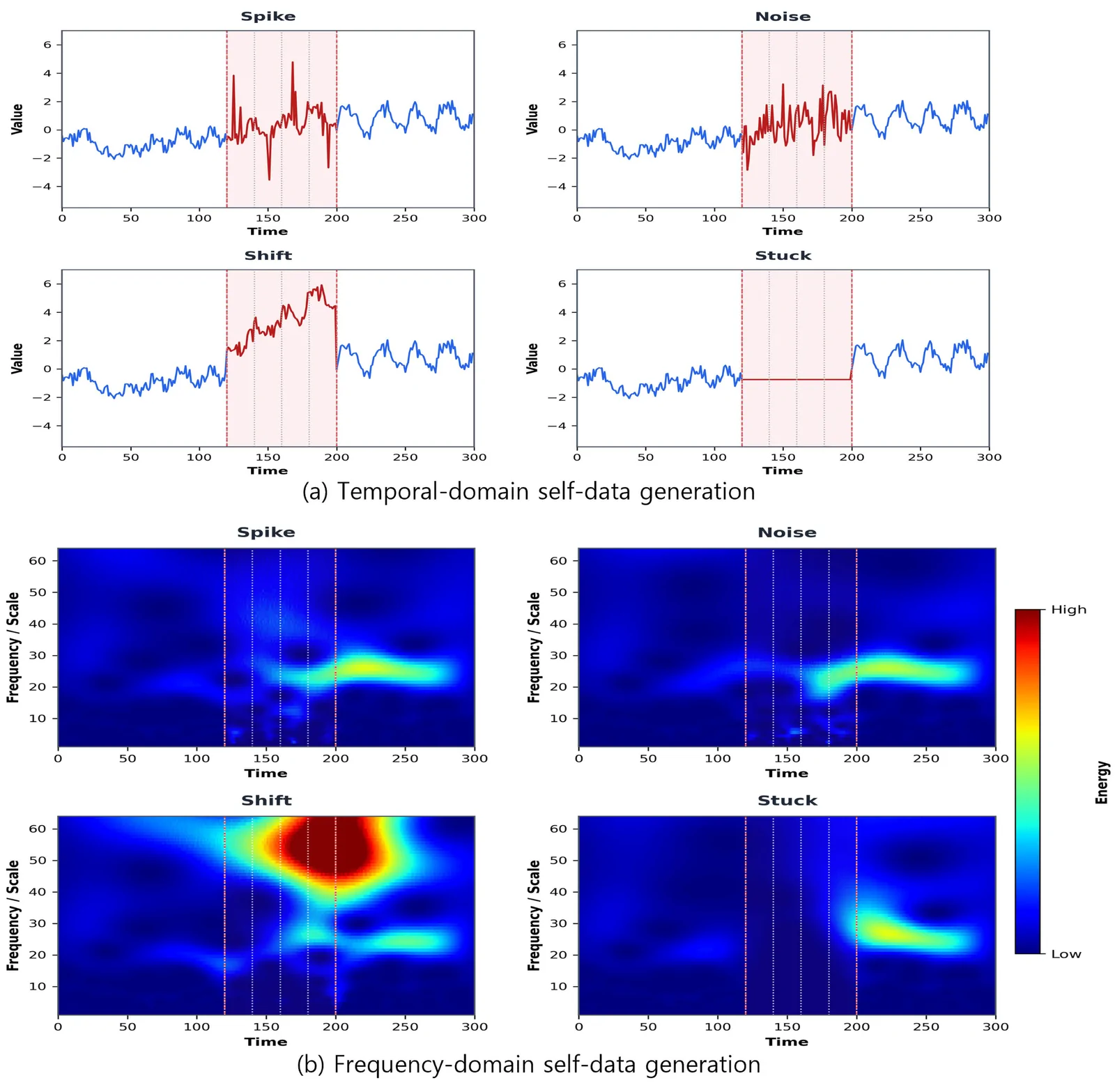
Examples of the domain-specific controlled synthetic perturbations used in SDG. (**a**) Temporal-domain perturbations consisting of spike, Gaussian noise, signal shift, stuck value, and random masking. (**b**) Representation-level perturbation of the frequency coefficients at the corresponding patch indices. The paired representations preserve patch-index correspondence rather than dynamic CWT transform consistency.

**Figure 6:**
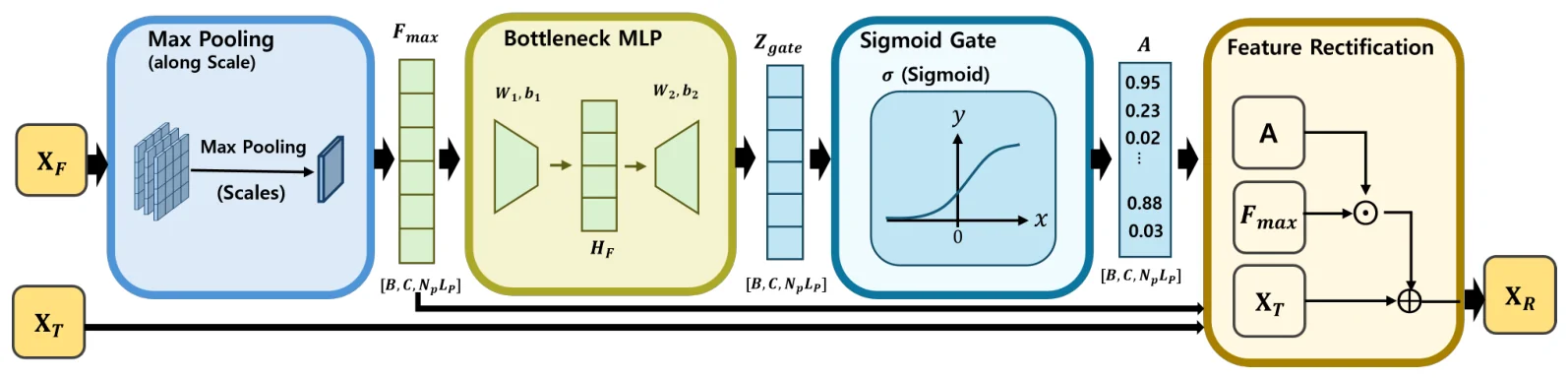
Structure of the Front Network. The rectification gate A is generated from $F_{\max}$ and used to integrate frequency-domain information into the temporal representation, producing $X_{R}$.

**Figure 7:**
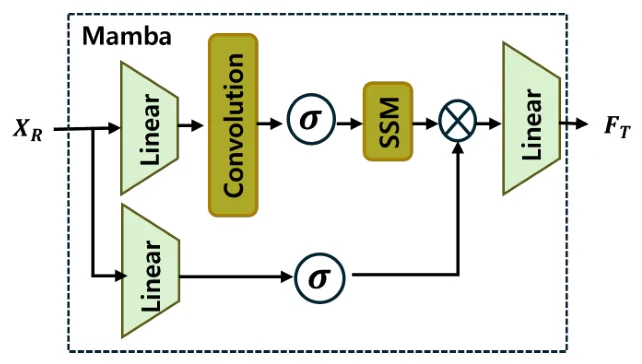
Structure of the Mamba block used in the Mamba temporal expert, following the selective state space model design of Mamba [15].

**Figure 8:**
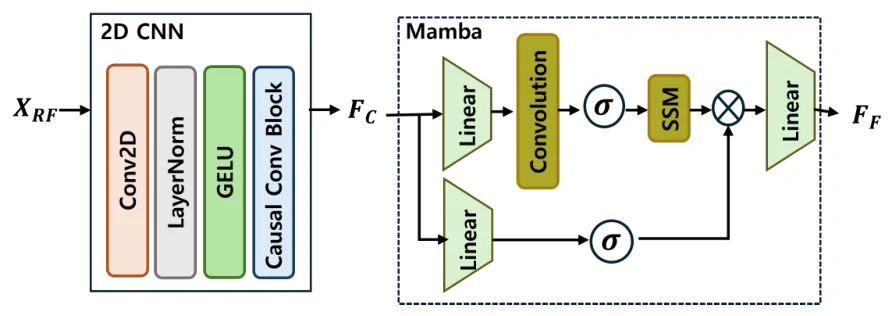
Architecture of the Mamba frequency expert. The rectified frequency-domain representation $X_{RF}$ is processed by the 2D CNN block to extract the intermediate frequency-domain feature $F_{C}$, which is then encoded by the Mamba block $M_{F}\left(\cdot \right)$ to generate the frequency-domain feature $F_{F}$.

**Figure 9:**
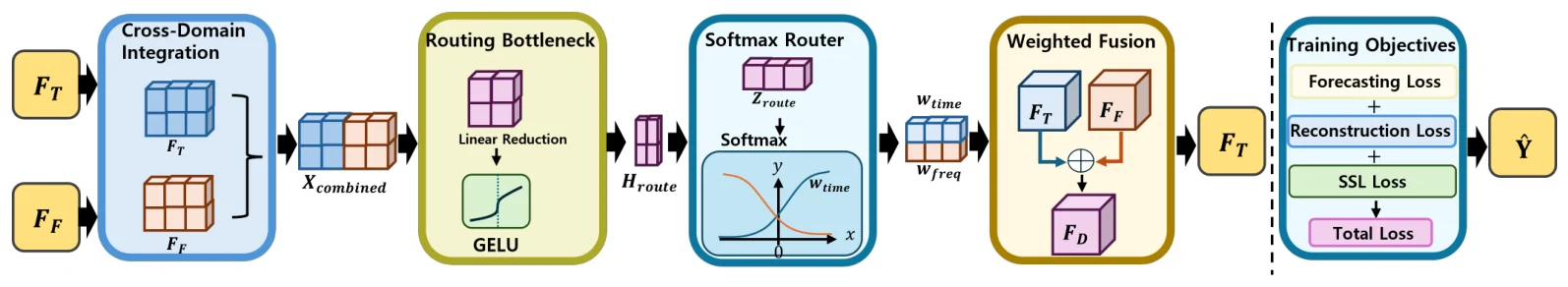
Structure of the Rear Network with the adaptive fusion mechanism. The temporal feature $F_{T}$ and frequency-domain feature $F_{F}$ are integrated through a routing bottleneck and softmax router to generate the fused forecasting representation $F_{D}$. The forecasting output $\hat{Y}$ is optimized using forecasting loss, reconstruction loss, and self-supervised contrastive loss.

**Figure 10:**
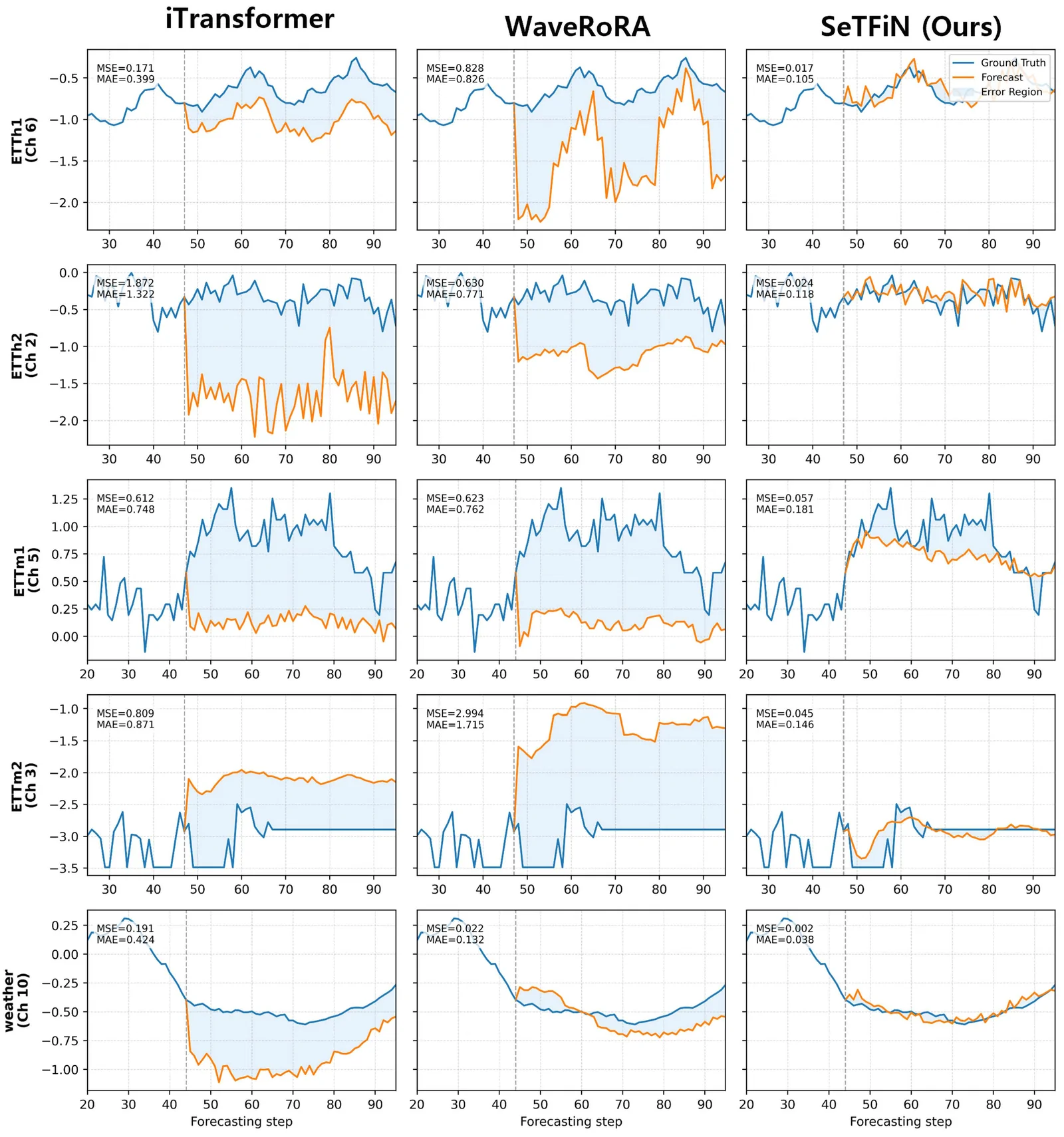
Qualitative forecasting comparison under the long-lookback forecasting setting (L=1440, H=96). The blue line denotes the ground truth, the orange line denotes the forecast, and the shaded area indicates the forecasting error region. The results are shown for five benchmark datasets and three representative models: iTransformer, WaveRoRA, and SeTFiN.

**Figure 11:**
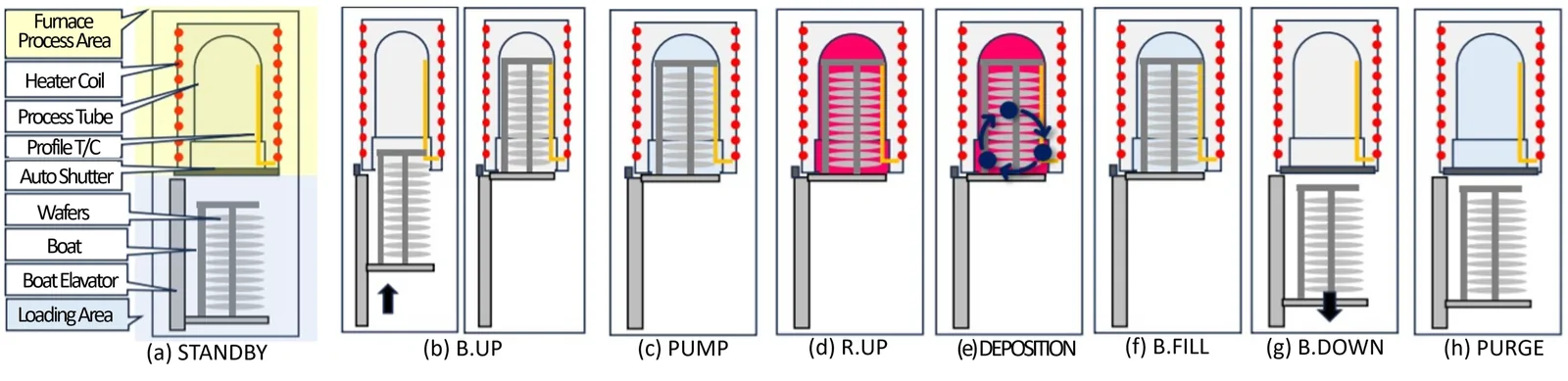
Eight-stage recipe-driven operation of the vertical LPCVD furnace used to collect the SFD: (**a**) standby and wafer loading; (**b**) boat elevation; (**c**) vacuum evacuation and leak inspection; (**d**) thermal ramp-up; (**e**) precursor delivery and thin-film deposition; (**f**) nitrogen backfill and chamber repressurization; (**g**) boat lowering and wafer unloading; and (**h**) post-purge and final atmospheric stabilization.

**Table 1:** Summary of the multivariate sensor time-series forecasting datasets used in this study.

No.	Dataset	Sensors (Dimensions)	Training Data	Validation Data	Test Data
1	ETTh1	7	8640	3216	3216
2	ETTh2	7	8640	3216	3216
3	ETTm1	7	34,560	11,856	11,856
4	ETTm2	7	34,560	11,856	11,856
5	Weather	21	36,887	5269	10,540
6	SFD	17	8416	2104	6312

**Table 2:** Long-term forecasting performance on five benchmark datasets under H∈{96, 192, 336, 720}. Results were evaluated on clean test sets. Lower values indicate better performance, and the best results are shown in bold.

Setting		SeTFiN (Ours)		WaveRoRA		iTransformer		PatchTST		Informer		DLinear		Autoformer	
Dataset	H	MSE	MAE	MSE	MAE	MSE	MAE	MSE	MAE	MSE	MAE	MSE	MAE	MSE	MAE
ETTh1	96	0.383	0.407	0.457	0.472	**0.377**	**0.402**	0.384	**0.402**	0.896	0.707	0.391	0.403	0.529	0.501
	192	0.437	0.435	0.512	0.505	**0.426**	0.433	0.432	**0.430**	1.110	0.818	0.443	0.436	0.558	0.516
	336	**0.468**	**0.453**	0.587	0.556	0.473	0.464	0.474	**0.453**	1.163	0.850	0.486	0.461	0.619	0.544
	720	**0.476**	**0.481**	0.719	0.661	0.525	0.521	0.554	0.521	1.294	0.925	0.514	0.509	0.676	0.586
	Avg.	**0.441**	**0.444**	0.569	0.549	0.450	0.455	0.461	0.452	1.116	0.825	0.459	0.452	0.596	0.537
ETTh2	96	**0.310**	**0.361**	0.786	0.652	0.343	0.372	0.323	0.375	1.881	1.136	0.344	0.396	0.411	0.449
	192	**0.384**	**0.406**	0.851	0.669	0.461	0.445	0.436	0.448	2.275	1.259	0.471	0.471	0.479	0.483
	336	**0.417**	**0.431**	1.248	0.863	0.543	0.496	0.582	0.515	2.412	1.306	0.585	0.535	0.478	0.485
	720	**0.431**	**0.447**	1.749	1.064	0.812	0.636	0.933	0.659	3.664	1.653	0.806	0.646	0.489	0.492
	Avg.	**0.386**	**0.411**	1.159	0.812	0.540	0.487	0.569	0.499	2.558	1.339	0.552	0.512	0.464	0.477
ETTm1	96	**0.328**	**0.366**	0.396	0.436	0.331	0.373	0.339	0.374	0.714	0.600	0.344	0.371	0.561	0.517
	192	**0.368**	**0.391**	0.433	0.455	0.378	0.407	0.382	0.401	0.784	0.642	0.382	**0.391**	0.755	0.596
	336	**0.401**	0.415	0.513	0.513	0.411	0.432	0.414	0.428	0.812	0.644	0.413	**0.413**	0.672	0.560
	720	**0.453**	**0.449**	0.537	0.519	0.466	0.465	0.474	0.467	0.913	0.704	0.472	0.450	0.664	0.558
	Avg.	**0.388**	**0.405**	0.470	0.481	0.397	0.419	0.402	0.418	0.806	0.648	0.403	0.406	0.663	0.558
ETTm2	96	**0.182**	**0.266**	0.265	0.345	0.197	0.280	0.202	0.283	0.909	0.741	0.193	0.291	0.268	0.357
	192	**0.247**	**0.309**	0.501	0.514	0.287	0.343	0.304	0.358	1.069	0.844	0.283	0.359	0.330	0.386
	336	**0.312**	**0.350**	0.597	0.563	0.374	0.384	0.360	0.394	1.296	0.933	0.371	0.417	0.389	0.431
	720	**0.433**	**0.423**	0.919	0.689	0.544	0.490	0.561	0.518	3.693	1.544	0.523	0.504	0.441	0.433
	Avg.	**0.294**	**0.337**	0.571	0.528	0.351	0.374	0.357	0.388	1.742	1.016	0.343	0.393	0.357	0.402
Weather	96	**0.161**	**0.207**	0.169	0.234	0.165	0.219	0.171	0.232	0.246	0.321	0.195	0.254	0.301	0.371
	192	0.210	**0.251**	0.220	0.282	**0.206**	0.259	0.212	0.271	0.302	0.357	0.236	0.293	0.428	0.456
	336	0.265	**0.292**	0.271	0.316	**0.258**	0.302	0.265	0.312	0.359	0.406	0.282	0.332	**0.258**	0.437
	720	0.345	**0.343**	0.339	0.365	**0.330**	0.350	0.332	0.363	0.403	0.435	0.346	0.383	0.447	0.449
	Avg.	0.245	**0.273**	0.250	0.299	**0.240**	0.283	0.245	0.295	0.328	0.380	0.265	0.316	0.359	0.428

**Table 3:** Long-lookback forecasting performance comparison under a fixed forecasting horizon of H=96 across five benchmark datasets. The lookback length *L* is varied from 720 to 5760. Lower MSE and MAE values indicate better forecasting performance, and the best results among the models included in this table are highlighted in **bold**.

Dataset	Model	L=720		L=1440		L=2880		L=5760		Avg.	
		MSE	MAE	MSE	MAE	MSE	MAE	MSE	MAE	MSE	MAE
ETTh1	SeTFiN (Ours)	0.411	**0.430**	**0.406**	**0.436**	0.498	**0.498**	0.750	**0.619**	**0.516**	**0.496**
	WaveRoRA	0.542	0.540	0.551	0.557	0.634	0.603	0.791	0.675	0.630	0.594
	iTransformer	**0.410**	0.443	0.441	0.476	**0.471**	0.499	**0.741**	0.646	**0.516**	0.516
ETTh2	SeTFiN (Ours)	**0.320**	**0.376**	**0.406**	**0.428**	**0.422**	**0.457**	**1.208**	**0.790**	**0.589**	**0.513**
	WaveRoRA	0.432	0.464	1.581	0.984	1.666	0.946	2.433	1.236	1.528	0.908
	iTransformer	0.610	0.560	0.915	0.723	1.572	0.950	2.007	1.107	1.276	0.835
ETTm1	SeTFiN (Ours)	0.309	0.365	0.339	**0.378**	**0.367**	**0.394**	**0.358**	**0.400**	**0.343**	**0.384**
	WaveRoRA	0.367	0.413	0.376	0.419	0.404	0.441	0.405	0.455	0.388	0.432
	iTransformer	**0.298**	**0.357**	**0.322**	0.385	0.386	0.436	0.414	0.466	0.355	0.411
ETTm2	SeTFiN (Ours)	**0.187**	**0.270**	**0.183**	**0.272**	**0.198**	**0.291**	**0.224**	**0.301**	**0.198**	**0.283**
	WaveRoRA	0.339	0.403	0.543	0.548	0.454	0.507	0.758	0.617	0.524	0.519
	iTransformer	0.196	0.285	0.263	0.329	0.337	0.383	0.546	0.515	0.336	0.378
Weather	SeTFiN (Ours)	**0.149**	**0.201**	**0.162**	**0.221**	**0.175**	**0.235**	**0.194**	**0.256**	**0.170**	**0.228**
	WaveRoRA	0.164	0.230	0.173	0.244	0.193	0.273	0.235	0.307	0.191	0.263
	iTransformer	0.150	0.207	0.163	0.233	0.197	0.276	0.237	0.315	0.186	0.258

**Table 4:** Effect of self-data generation on forecasting performance across five benchmark datasets under the L=H=96 setting. Lower MSE and MAE values indicate better forecasting performance, and the best results are highlighted in **bold**.

Variant	ETTh1		ETTh2		ETTm1		ETTm2		Weather		Avg.	
	MSE	MAE	MSE	MAE	MSE	MAE	MSE	MAE	MSE	MAE	MSE	MAE
w/o SDG	0.392	0.413	0.318	0.368	0.338	0.374	0.190	0.277	0.169	0.220	0.282	0.330
With SDG	**0.383**	**0.407**	**0.310**	**0.361**	**0.328**	**0.366**	**0.182**	**0.266**	**0.161**	**0.207**	**0.273**	**0.321**

**Table 5:** Ablation study of temporal and frequency-domain components under the short-horizon setting (L=720, H=16). Self-data generation was enabled to match the final SeTFiN training condition. Lower MSE and MAE values indicate better forecasting performance, and the best results are highlighted in bold.

Variant	ETTh1		ETTh2		ETTm1		ETTm2		Weather		Avg. MSE	Avg. MAE
	MSE	MAE	MSE	MAE	MSE	MAE	MSE	MAE	MSE	MAE		
w/o Temporal	0.354	0.391	0.184	0.285	0.158	**0.250**	**0.085**	**0.181**	0.082	0.117	0.173	0.245
w/o Frequency	0.354	0.388	0.173	0.272	0.156	**0.250**	0.086	0.183	0.082	0.115	0.170	0.242
SeTFiN (Ours)	**0.348**	**0.387**	**0.168**	**0.269**	**0.154**	**0.250**	**0.085**	**0.181**	**0.080**	**0.107**	**0.167**	**0.239**

**Table 6:** Eight-stage recipe-driven operation of the vertical LPCVD furnace used for SFD collection.

Index	Stage Name	Detailed Recipe Steps	Physical Description
(a)	STANDBY	STANDBY	Wafer loading under atmospheric conditions
(b)	B.UP	B.UP	Boat elevation into the process tube
(c)	PUMP	S.P, M.P, L.CHK	Vacuum evacuation and leak inspection
(d)	R.UP	R.UP	Thermal ramp-up to the target setpoint
(e)	DEPOSITION	N-EVA1, CLOSE1, SI-FL1, SI-EVA1, N-FL1	Precursor delivery and thin-film deposition
(f)	B.FILL	B.FILL1, B.FILL2, B.FILL3, B.FILL4	Inert nitrogen backfill and chamber repressurization
(g)	B.DOWN	B.DOWN	Boat lowering and wafer unloading
(h)	PURGE	VAC_LOOP1∼B.FILL4	Post-purge and final atmospheric stabilization

**Table 7:** Sensor variables included in the SFD.

Category	Variables	Number
Gas	GAS_DCS, GAS_F2, GAS_N2_1, GAS_N2_2, GAS_N2_3, GAS_N2_4, GAS_N2_LOADING_AREA, GAS_N2_NR, and GAS_NH3	9
Pressure	PRESSURE_APC_ANGLE, PRESSURE_VG1, and PRESSURE_VG2	3
Temperature	TEMP_PROFILE_1, TEMP_PROFILE_2, TEMP_PROFILE_3, TEMP_PROFILE_4, and TEMP_PROFILE_5	5
Total		17

**Table 8:** Long-look forecasting performance on the industrial SFD under the clean evaluation setting. Results are reported using random seed 2023 with L=96 and H∈{96, 192, 336, 720}. Lower values indicate better performance, and the best results are highlighted in **bold**.

*H*	SeTFiN (Ours)		WaveRoRA		iTransformer		PatchTST		Informer		DLinear		Autoformer	
	MSE	MAE	MSE	MAE	MSE	MAE	MSE	MAE	MSE	MAE	MSE	MAE	MSE	MAE
96	**3.835**	**0.335**	76.314	4.515	69.131	3.745	64.359	3.605	98.473	5.173	4.993	0.737	6.270	0.789
192	**6.643**	**0.552**	80.337	4.456	76.390	4.059	78.747	4.122	97.832	5.120	8.452	1.111	9.507	0.997
336	**10.452**	**0.801**	92.344	4.843	85.812	4.388	84.289	4.356	98.804	5.134	12.595	1.489	13.856	1.257
720	23.580	**1.511**	91.914	4.898	87.333	4.505	86.978	4.511	95.874	5.057	**20.349**	2.097	24.611	1.819
Avg.	**11.127**	**0.800**	85.227	4.678	79.666	4.174	78.593	4.148	97.746	5.121	11.597	1.358	13.561	1.215

**Table 9:** Long-lookback forecasting performance on the SFD under a fixed forecasting horizon of H=96. Lower values indicate better performance, and the best results are highlighted in **bold**.

Model	Metric	L=720	L=1440	L=2880	L=5760	Avg.
SeTFiN (Ours)	MSE	**5.652**	**6.769**	**13.327**	**45.829**	**17.894**
	MAE	**0.703**	**0.893**	**1.554**	**3.930**	**1.770**
WaveRoRA	MSE	87.591	92.124	97.287	98.569	93.893
	MAE	4.854	4.964	5.128	5.212	5.040
iTransformer	MSE	84.241	87.085	92.881	97.004	90.303
	MAE	4.325	4.480	4.799	5.072	4.669

**Table 10:** Forecasting performance of SeTFiN with and without SDG under five controlled synthetic test-time degradation conditions on the industrial SFD (L=720, H=16). Results are reported using random seed 2023. The best result for each metric and degradation condition is highlighted in **bold**.

Variant	Spike		Noise		Shift		Stuck		Masking	
	MSE	MAE	MSE	MAE	MSE	MAE	MSE	MAE	MSE	MAE
w/o SDG	2.023	**0.439**	1.840	**0.386**	1.801	**0.334**	1.864	**0.326**	12.668	1.270
SeTFiN	**1.973**	0.473	**1.790**	0.414	**1.710**	0.347	**1.762**	0.338	**12.528**	**1.201**

**Figure A1:**
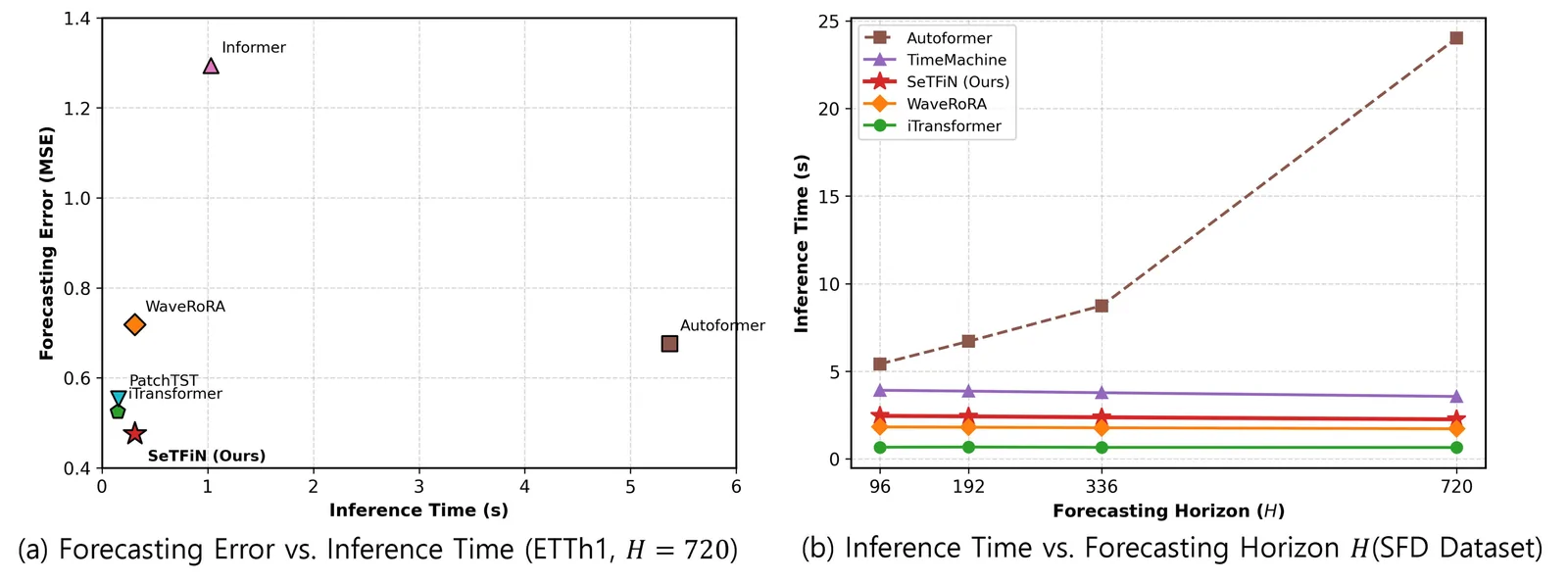
Inference efficiency analysis of SeTFiN and baseline models. (**a**) Forecasting error (MSE) versus total test-set inference time on ETTh1 (L=96, H=720). (**b**) Total test-set inference time across forecasting horizons (H∈{96, 192, 336, 720}) on the industrial SFD.

## Data Availability

Publicly available datasets were analyzed in this study. The data and repository links are explicitly mentioned within the manuscript.

## Appendix A. Multihorizon Forecasting Benchmarks and Configuration-Level Statistical Analysis

This appendix presents extended multihorizon forecasting results under L=96 and H∈{96, 192, 336, 720}. Table A1 reports the mean and sample standard deviation over three independent runs using random seeds 2023, 2024, and 2025 for five public benchmark datasets and the industrial SFD. Table A2 provides exploratory configuration-level comparisons based on the three-seed mean MSE for each dataset–horizon setting.

For the public benchmarks, each paired observation comprises the three-seed mean MSE values of SeTFiN and a baseline evaluated under the same dataset–horizon configuration, resulting in 20 matched pairs. Pairing is therefore performed across configurations rather than across individual random-seed runs. These analyses characterize the direction and magnitude of performance differences across the evaluated configurations but do not establish seed-level statistical significance for individual forecasting tasks. Because only four horizon configurations are available for the SFD, the SFD results are summarized descriptively without drawing inferential conclusions from this small sample.

**Table A1:** Multihorizon forecasting performance on public benchmarks and the SFD under a fixed lookback length of L=96. Results are reported as the mean ± sample standard deviation over three independent runs (random seeds 2023, 2024, and 2025). Lower values indicate better performance, and the best results are highlighted in bold.

Setting		SeTFiN (Ours)		TimeMachine		WaveRoRA		iTransformer		PatchTST		Informer	
Dataset	H	MSE	MAE	MSE	MAE	MSE	MAE	MSE	MAE	MSE	MAE	MSE	MAE
ETTh1	96	0.389±0.005	0.409±0.004	0.385±0.006	0.399±0.003	0.475±0.024	0.486±0.017	0.377±0.001	0.402±0.000	0.384±0.000	0.402±0.001	1.083±0.319	0.786±0.134
	192	0.438±0.001	0.436±0.002	0.434±0.006	0.429±0.003	0.514±0.009	0.509±0.009	0.430±0.006	0.437±0.007	0.434±0.002	0.431±0.001	1.054±0.114	0.780±0.063
	336	0.473±0.005	0.455±0.002	0.479±0.005	0.450±0.002	0.571±0.014	0.544±0.011	0.474±0.001	0.466±0.002	0.480±0.007	0.459±0.007	1.174±0.012	0.843±0.008
	720	0.482±0.013	0.481±0.006	0.482±0.013	0.472±0.009	0.723±0.007	0.662±0.001	0.526±0.003	0.520±0.001	0.584±0.031	0.538±0.016	1.292±0.005	0.924±0.002
ETTh2	96	0.307±0.003	0.359±0.003	0.293±0.003	0.344±0.001	0.732±0.089	0.627±0.049	0.341±0.006	0.374±0.006	0.326±0.004	0.379±0.004	1.845±0.034	1.107±0.031
	192	0.387±0.004	0.409±0.005	0.371±0.007	0.393±0.003	0.857±0.027	0.676±0.018	0.456±0.005	0.442±0.003	0.445±0.014	0.448±0.001	2.146±0.117	1.202±0.050
	336	0.419±0.004	0.434±0.003	0.418±0.006	0.428±0.003	1.216±0.045	0.853±0.024	0.536±0.007	0.492±0.004	0.547±0.032	0.502±0.013	2.327±0.074	1.271±0.031
	720	0.430±0.002	0.448±0.001	0.426±0.005	0.443±0.002	1.848±0.185	1.100±0.062	0.849±0.047	0.647±0.011	0.836±0.084	0.636±0.021	2.915±0.649	1.449±0.176
ETTm1	96	0.332±0.004	0.366±0.002	0.322±0.004	0.358±0.003	0.401±0.006	0.436±0.003	0.334±0.002	0.375±0.002	0.344±0.005	0.377±0.003	0.681±0.030	0.584±0.016
	192	0.370±0.006	0.391±0.003	0.367±0.004	0.383±0.002	0.439±0.006	0.461±0.005	0.376±0.003	0.404±0.003	0.380±0.003	0.399±0.004	0.752±0.034	0.633±0.012
	336	0.395±0.006	0.412±0.004	0.400±0.004	0.406±0.001	0.510±0.006	0.513±0.006	0.410±0.003	0.430±0.003	0.416±0.002	0.429±0.003	0.822±0.009	0.648±0.006
	720	0.455±0.003	0.449±0.002	0.468±0.009	0.445±0.003	0.554±0.019	0.535±0.017	0.464±0.003	0.463±0.003	0.474±0.008	0.467±0.009	0.919±0.006	0.705±0.002
ETTm2	96	0.182±0.002	0.265±0.002	0.177±0.001	0.258±0.001	0.267±0.010	0.349±0.014	0.193±0.004	0.277±0.003	0.197±0.004	0.281±0.003	0.668±0.214	0.629±0.102
	192	0.248±0.002	0.309±0.002	0.241±0.001	0.301±0.002	0.425±0.070	0.465±0.046	0.277±0.010	0.338±0.009	0.303±0.017	0.355±0.009	1.037±0.051	0.826±0.021
	336	0.313±0.003	0.351±0.002	0.300±0.003	0.338±0.002	0.608±0.046	0.568±0.027	0.373±0.002	0.391±0.008	0.361±0.017	0.395±0.015	1.267±0.034	0.918±0.013
	720	0.430±0.007	0.421±0.005	0.401±0.007	0.397±0.003	0.975±0.145	0.728±0.079	0.544±0.004	0.489±0.002	0.722±0.142	0.537±0.020	3.209±0.429	1.422±0.116
Weather	96	0.160±0.002	0.207±0.001	0.173±0.004	0.215±0.002	0.170±0.002	0.232±0.002	0.165±0.000	0.219±0.001	0.172±0.001	0.232±0.001	0.253±0.011	0.322±0.004
	192	0.212±0.002	0.253±0.002	0.220±0.003	0.257±0.001	0.218±0.002	0.279±0.003	0.206±0.001	0.259±0.001	0.214±0.003	0.275±0.005	0.299±0.011	0.357±0.012
	336	0.268±0.002	0.294±0.002	0.276±0.003	0.297±0.002	0.275±0.005	0.323±0.008	0.257±0.001	0.301±0.001	0.264±0.002	0.313±0.004	0.359±0.020	0.404±0.018
	720	0.345±0.002	0.344±0.002	0.351±0.004	0.346±0.002	0.347±0.008	0.370±0.004	0.331±0.002	0.353±0.003	0.331±0.001	0.362±0.002	0.415±0.013	0.440±0.007
SFD	96	4.464±0.545	0.423±0.076	4.621±0.053	0.477±0.008	77.138±0.722	4.428±0.082	70.301±1.026	3.796±0.045	65.828±1.546	3.662±0.053	101.652±3.394	5.264±0.095
	192	7.468±0.716	0.665±0.098	8.061±0.010	0.760±0.004	82.665±2.769	4.667±0.203	77.907±1.315	4.117±0.050	79.666±0.796	4.157±0.030	99.777±1.935	5.228±0.096
	336	11.012±0.485	0.864±0.055	13.016±0.099	1.112±0.015	93.247±0.797	4.880±0.033	87.141±1.150	4.441±0.046	85.775±1.308	4.404±0.042	100.742±1.754	5.247±0.126
	720	23.566±0.020	1.528±0.014	24.463±0.114	1.721±0.012	93.370±1.855	4.962±0.069	88.976±1.430	4.556±0.044	88.388±1.221	4.559±0.041	97.296±1.357	5.109±0.045

**Table A2:** Exploratory configuration-level comparisons between SeTFiN and baseline models across multihorizon forecasting settings (L=96, H∈{96, 192, 336, 720}). For the public benchmarks, paired observations correspond to the three-seed mean MSE values obtained under matched dataset–horizon configurations. The SFD results are summarized descriptively because only four horizon configurations are available.

Opponent Baseline	Public Benchmarks (20 Dataset–Horizon Configurations)					Industrial SFD (4 Horizon Configurations)	
	Win/Tie/Loss	Paired t (p)	Wilcoxon (p)	Cohen’s $d_{z}$	Exploratory Interpretation	Win/Tie/Loss	Descriptive Summary
TimeMachine	4/0/16	0.007	<0.001	−0.67	Lower TimeMachine MSE in 16 of 20 configurations	2/0/2	Mixed: each model lower MSE in two of four settings
iTransformer	15/0/5	0.054	0.015	+0.46	Lower SeTFiN MSE in 15 of 20 configurations	4/0/0	Lower SeTFiN MSE in all four configurations
PatchTST	16/0/4	0.024	0.001	+0.55	Lower SeTFiN MSE in 16 of 20 configurations	4/0/0	Lower SeTFiN MSE in all four configurations
WaveRoRA	20/0/0	0.004	<0.001	+0.73	Lower SeTFiN MSE in all 20 configurations	4/0/0	Lower SeTFiN MSE in all four configurations
Informer	20/0/0	<0.001	<0.001	+1.09	Lower SeTFiN MSE in all 20 configurations	4/0/0	Lower SeTFiN MSE in all four configurations

Note: Win/tie/loss is reported from the perspective of SeTFiN. For the public benchmarks, two-sided paired *t*-tests and two-sided Wilcoxon signed-rank tests were applied exploratorily to the three-seed mean MSE values from 20 matched dataset–horizon configurations. A positive Cohen’s *d_z_* indicates lower MSE for SeTFiN, whereas a negative value indicates lower MSE for the baseline. The unit of analysis is the dataset–horizon configuration rather than an individual random-seed run. Therefore, these analyses do not establish seed-level statistical significance for individual forecasting tasks. Because only four configurations are available for the SFD, no inferential tests or effect sizes are reported for the SFD comparisons.

## Appendix B. Long-Lookback Forecasting Benchmarks and Configuration-Level Statistical Analysis

This appendix presents long-lookback forecasting evaluations under input lengths L∈{720, 1440, 2880, 5760} with a fixed forecasting horizon of H=96. Table A3 reports the mean and sample standard deviation over three independent runs using random seeds 2023, 2024, and 2025 across five public benchmark datasets and the industrial SFD. Table A4 (on the following page) provides exploratory configuration-level comparisons based on the three-seed mean MSE for each dataset–lookback setting.

For the public benchmarks, each paired observation comprises the three-seed mean MSE values of SeTFiN and a baseline evaluated under the same dataset–lookback configuration, resulting in 20 matched pairs. Pairing is therefore performed across configurations rather than across individual random-seed runs. These analyses characterize the direction and magnitude of performance differences across the evaluated configurations but do not establish seed-level statistical significance for individual forecasting tasks. Because only four lookback configurations are available for the SFD, the SFD results are summarized descriptively without drawing inferential conclusions from this small sample. The individual seed-level results underlying the comparison between SeTFiN and TimeMachine are reported in Table A5.

**Table A3:** Long-lookback forecasting performance under a fixed forecasting horizon of H=96 across extended input lengths L∈{720, 1440, 2880, 5760}. Results are reported as the mean ± sample standard deviation over three independent runs (random seeds 2023, 2024, and 2025). Lower values indicate better performance, and the best results are highlighted in bold.

Setting		SeTFiN (Ours)		TimeMachine		WaveRoRA		iTransformer	
Dataset	L	MSE	MAE	MSE	MAE	MSE	MAE	MSE	MAE
ETTh1	720	0.402±0.008	0.427±0.003	0.387±0.001	0.415±0.001	0.525±0.019	0.536±0.010	0.405±0.006	0.441±0.003
	1440	0.408±0.004	0.436±0.003	0.391±0.006	0.420±0.005	0.557±0.063	0.555±0.037	0.432±0.008	0.468±0.007
	2880	0.485±0.013	0.490±0.008	0.409±0.001	0.437±0.001	0.606±0.027	0.580±0.020	0.480±0.008	0.503±0.005
	5760	0.740±0.013	0.619±0.008	0.572±0.025	0.552±0.016	0.786±0.011	0.676±0.006	0.745±0.038	0.643±0.022
	Avg.	0.509±0.007	0.493±0.003	0.439±0.006	0.456±0.004	0.619±0.021	0.587±0.011	0.516±0.010	0.514±0.005
ETTh2	720	0.325±0.009	0.380±0.007	0.287±0.003	0.346±0.001	0.611±0.202	0.578±0.134	0.604±0.029	0.552±0.017
	1440	0.413±0.026	0.436±0.014	0.293±0.004	0.357±0.002	1.907±0.429	1.075±0.125	0.938±0.058	0.732±0.022
	2880	0.500±0.068	0.499±0.036	0.357±0.025	0.417±0.019	1.732±0.175	0.987±0.080	1.537±0.096	0.941±0.024
	5760	1.040±0.152	0.769±0.022	0.728±0.037	0.655±0.016	2.280±0.318	1.200±0.106	2.015±0.050	1.118±0.017
	Avg.	0.569±0.020	0.521±0.007	0.416±0.015	0.444±0.008	1.632±0.090	0.960±0.045	1.274±0.042	0.836±0.010
ETTm1	720	0.307±0.002	0.361±0.004	0.301±0.005	0.353±0.003	0.353±0.016	0.406±0.008	0.294±0.004	0.355±0.003
	1440	0.337±0.006	0.378±0.004	0.308±0.002	0.360±0.002	0.378±0.003	0.420±0.001	0.316±0.005	0.374±0.010
	2880	0.349±0.016	0.390±0.004	0.322±0.006	0.373±0.004	0.394±0.009	0.441±0.001	0.375±0.010	0.423±0.012
	5760	0.381±0.020	0.411±0.010	0.340±0.006	0.388±0.004	0.413±0.007	0.464±0.008	0.408±0.006	0.455±0.009
	Avg.	0.343±0.002	0.385±0.002	0.318±0.004	0.368±0.002	0.385±0.004	0.433±0.001	0.348±0.006	0.402±0.008
ETTm2	720	0.185±0.002	0.271±0.001	0.171±0.004	0.260±0.002	0.300±0.034	0.375±0.024	0.198±0.005	0.282±0.004
	1440	0.183±0.004	0.272±0.001	0.175±0.002	0.268±0.002	0.464±0.078	0.501±0.048	0.258±0.004	0.323±0.006
	2880	0.223±0.049	0.304±0.026	0.171±0.003	0.270±0.003	0.515±0.054	0.545±0.034	0.330±0.015	0.379±0.009
	5760	0.212±0.012	0.297±0.005	0.191±0.003	0.286±0.002	0.769±0.039	0.630±0.019	0.513±0.046	0.503±0.040
	Avg.	0.201±0.012	0.286±0.007	0.177±0.002	0.271±0.001	0.512±0.011	0.513±0.005	0.325±0.012	0.372±0.009
Weather	720	0.149±0.000	0.200±0.001	0.156±0.002	0.207±0.001	0.163±0.001	0.227±0.003	0.148±0.002	0.204±0.004
	1440	0.157±0.004	0.214±0.006	0.165±0.003	0.222±0.003	0.175±0.007	0.242±0.008	0.158±0.004	0.222±0.009
	2880	0.165±0.008	0.227±0.007	0.169±0.005	0.230±0.004	0.196±0.003	0.272±0.005	0.186±0.010	0.256±0.018
	5760	0.186±0.009	0.250±0.007	0.192±0.004	0.258±0.003	0.241±0.007	0.320±0.012	0.230±0.006	0.305±0.009
	Avg.	0.163±0.008	0.220±0.009	0.170±0.002	0.229±0.002	0.194±0.004	0.265±0.004	0.180±0.005	0.247±0.010
SFD	720	5.618±0.127	0.675±0.025	6.660±0.535	0.858±0.078	91.299±3.212	4.956±0.091	84.482±0.215	4.331±0.005
	1440	6.964±0.187	0.906±0.013	7.558±0.245	1.046±0.035	91.904±1.019	4.993±0.047	87.025±0.314	4.479±0.010
	2880	12.829±0.813	1.545±0.048	9.071±0.504	1.214±0.067	96.830±0.397	5.122±0.013	93.059±0.157	4.788±0.010
	5760	53.170±7.248	4.165±0.223	24.078±0.320	2.591±0.017	98.777±0.473	5.212±0.008	95.727±1.106	5.057±0.014
	Avg.	19.645±1.691	1.822±0.047	11.842±0.152	1.427±0.017	94.702±0.781	5.071±0.030	90.073±0.211	4.664±0.005

### Configuration-Level Analysis and Seed-Level Results

**Table A4:** Exploratory configuration-level comparisons between SeTFiN and baseline models under extended-lookback forecasting settings (L∈{720, 1440, 2880, 5760}, H=96). For the public benchmarks, paired observations correspond to the three-seed mean MSE values obtained under matched dataset–lookback configurations. The SFD results are summarized descriptively because only four lookback configurations are available.

Opponent Baseline	Public Benchmarks (20 Dataset–Lookback Configurations)					Industrial SFD (4 Lookback Configurations)	
	Win/Tie/Loss	Paired t (p)	Wilcoxon (p)	Cohen’s $d_{z}$	Exploratory Interpretation	Win/Tie/Loss	Descriptive Summary
iTransformer	16/0/4	0.026	0.001	+0.54	Lower SeTFiN MSE in 16 of 20 configurations	4/0/0	Lower SeTFiN MSE in all four configurations
WaveRoRA	20/0/0	0.007	<0.001	+0.68	Lower SeTFiN MSE in all 20 configurations	4/0/0	Lower SeTFiN MSE in all four configurations
TimeMachine	4/0/16	0.007	<0.001	−0.67	Lower TimeMachine MSE in 16 of 20 configurations	2/0/2	Mixed: each model achieved lower MSE in two of four settings

Note: Win/tie/loss is reported from the perspective of SeTFiN. For the public benchmarks, two-sided paired *t*-tests and two-sided Wilcoxon signed-rank tests were applied exploratorily to the three-seed mean MSE values from 20 matched dataset–lookback configurations. A positive Cohen’s $d_{z}$ indicates lower MSE for SeTFiN, whereas a negative value indicates lower MSE for the baseline. The unit of analysis is the dataset–lookback configuration rather than an individual random-seed run. Therefore, these analyses do not establish seed-level statistical significance for individual forecasting tasks. Because only four configurations are available for the SFD, no inferential tests or effect sizes are reported for the SFD comparisons.

To document the run-to-run variation underlying the long-lookback comparisons, Table A5 reports the individual MSE and MAE results obtained using random seeds 2023, 2024, and 2025 for SeTFiN and TimeMachine across all six datasets under L∈{720, 1440, 2880, 5760} and H=96. These seed-level values are provided for transparency and are not treated as independent observations in the configuration-level statistical analysis reported in Table A4.

**Table A5:** Seed-level long-lookback forecasting results for SeTFiN and TimeMachine under H=96. Individual MSE and MAE values are reported for random seeds 2023, 2024, and 2025 across six datasets and input lengths L∈{720, 1440, 2880, 5760}.

Setting		Seed 2023				Seed 2024				Seed 2025			
Dataset	L	SeTFiN		TimeMachine		SeTFiN		TimeMachine		SeTFiN		TimeMachine	
		MSE	MAE	MSE	MAE	MSE	MAE	MSE	MAE	MSE	MAE	MSE	MAE
ETTh1	720	0.411	0.430	0.388	0.415	0.399	0.427	0.386	0.414	0.395	0.424	0.387	0.415
	1440	0.406	0.436	0.388	0.417	0.413	0.439	0.398	0.426	0.406	0.433	0.386	0.417
	2880	0.498	0.498	0.409	0.438	0.473	0.482	0.409	0.438	0.484	0.490	0.408	0.436
	5760	0.750	0.619	0.549	0.536	0.745	0.626	0.569	0.553	0.726	0.611	0.599	0.567
ETTh2	720	0.320	0.376	0.289	0.346	0.319	0.375	0.283	0.345	0.335	0.388	0.288	0.347
	1440	0.406	0.428	0.291	0.355	0.442	0.452	0.291	0.357	0.391	0.428	0.298	0.359
	2880	0.422	0.457	0.386	0.439	0.526	0.522	0.345	0.408	0.551	0.518	0.340	0.405
	5760	1.208	0.790	0.771	0.674	0.912	0.746	0.709	0.646	1.000	0.771	0.705	0.646
ETTm1	720	0.309	0.365	0.303	0.354	0.306	0.360	0.296	0.350	0.306	0.358	0.305	0.355
	1440	0.339	0.378	0.309	0.361	0.342	0.382	0.309	0.361	0.331	0.374	0.305	0.358
	2880	0.367	0.394	0.326	0.376	0.337	0.387	0.316	0.368	0.343	0.390	0.325	0.374
	5760	0.358	0.400	0.345	0.391	0.397	0.417	0.334	0.384	0.387	0.416	0.341	0.389
ETTm2	720	0.187	0.270	0.174	0.262	0.183	0.272	0.167	0.259	0.185	0.272	0.171	0.260
	1440	0.183	0.272	0.174	0.267	0.187	0.273	0.178	0.270	0.180	0.272	0.174	0.268
	2880	0.198	0.291	0.174	0.272	0.191	0.286	0.171	0.271	0.279	0.334	0.168	0.266
	5760	0.224	0.301	0.194	0.285	0.201	0.292	0.188	0.284	0.210	0.299	0.192	0.288
Weather	720	0.149	0.201	0.155	0.207	0.149	0.200	0.155	0.206	0.149	0.200	0.158	0.208
	1440	0.162	0.221	0.168	0.225	0.155	0.210	0.164	0.221	0.155	0.211	0.163	0.220
	2880	0.175	0.235	0.167	0.228	0.160	0.224	0.174	0.235	0.161	0.223	0.165	0.228
	5760	0.194	0.256	0.192	0.257	0.177	0.242	0.196	0.261	0.187	0.251	0.189	0.255
SFD	720	5.652	0.703	6.502	0.823	5.478	0.661	7.256	0.947	5.725	0.660	6.221	0.804
	1440	6.769	0.893	7.338	1.011	6.979	0.907	7.514	1.048	7.143	0.918	7.822	1.080
	2880	13.327	1.554	9.651	1.291	13.269	1.587	8.732	1.172	11.890	1.493	8.830	1.178
	5760	45.829	3.930	24.445	2.601	53.360	4.190	23.932	2.601	60.321	4.374	23.856	2.571

## Appendix C. Controlled Synthetic Degradation and Component Ablations

This appendix reports complementary ablations of SeTFiN. Table A6 evaluates the temporal and frequency experts. Table A7 and Table A8 examine SDG, architectural components, and auxiliary losses on the industrial SFD. Table A9 presents matched same-backbone controls under controlled synthetic SFD degradations.

Table A8 shows that the temporal expert had the largest architectural impact. Removing SDG caused the largest degradation-related error increase among the remaining components, whereas removing the SSL loss did not increase error in this setting.

Table A9 shows that the results depend on the training perturbation, test-time degradation, and metric. Full SDG achieved the lowest MSE under spike, noise, and shift, whereas simple masking was best under clean and stuck-value conditions. Alignment reduced MSE in four of five degradation conditions relative to unaligned SDG, but unaligned SDG performed best under random masking.

**Table A6:** Ablation study of the temporal and frequency experts across five benchmark datasets (L=720, H=16). Results are reported as the mean ± sample standard deviation over three independent runs (random seeds 2023, 2024, and 2025). Lower values indicate better performance, and the best results are highlighted in bold.

Variant	ETTh1		ETTh2		ETTm1		ETTm2		Weather		Avg.	
	MSE	MAE	MSE	MAE	MSE	MAE	MSE	MAE	MSE	MAE	MSE	MAE
w/o Temporal	0.352±0.003	0.389±0.002	0.186±0.002	0.287±0.003	0.160±0.003	0.252±0.002	0.085±0.000	0.181±0.001	0.081±0.001	0.112±0.004	0.173±0.001	0.244±0.001
w/o Frequency	0.354±0.007	0.389±0.005	0.181±0.008	0.281±0.009	0.159±0.003	0.252±0.003	0.085±0.002	0.182±0.002	0.081±0.002	0.110±0.004	0.172±0.002	0.243±0.002
**SeTFiN (Ours)**	0.350±0.003	0.389±0.001	0.174±0.006	0.274±0.007	0.157±0.006	0.251±0.004	0.084±0.000	0.182±0.001	0.080±0.001	0.108±0.001	0.170±0.003	0.241±0.002

**Table A7:** Forecasting performance of SeTFiN with and without Self-Data Generation (SDG) under five controlled synthetic sensor-degradation conditions on the industrial SFD dataset (L=720, H=16). Results are reported as the mean ± sample standard deviation over three independent runs (random seeds 2023, 2024, and 2025). Best results in bold.

Variant	Spike		Noise		Shift		Stuck		Masking	
	MSE	MAE	MSE	MAE	MSE	MAE	MSE	MAE	MSE	MAE
w/o SDG	1.623±0.347	0.444±0.011	1.422±0.364	0.383±0.008	1.291±0.442	0.294±0.035	1.286±0.501	0.277±0.043	11.444±1.068	1.252±0.019
**With SDG**	1.524±0.397	0.424±0.053	1.317±0.419	0.356±0.061	1.260±0.403	0.296±0.059	1.257±0.445	0.280±0.062	10.837±1.465	1.100±0.088

**Table A8:** Comprehensive component and auxiliary-loss ablation study of SeTFiN on the industrial SFD dataset (L=720, H=16). Results are reported as the mean ± sample standard deviation across three random seeds (2023, 2024, and 2025).

Variant	Clean MSE	Clean MAE	Degraded MSE	Degraded MAE
Architectural and SDG ablations				
SeTFiN (Full)	1.084±0.105	0.273±0.019	1.519±0.144	0.357±0.026
w/o SDG	1.207±0.416	0.293±0.102	4.891±0.217	0.745±0.026
w/o CDFR	1.350±0.199	0.332±0.034	1.993±0.318	0.443±0.045
w/o Adaptive Fusion	1.232±0.030	0.317±0.010	1.650±0.099	0.389±0.020
w/o Temporal Expert	17.615±0.381	1.652±0.011	17.513±0.288	1.654±0.011
w/o Frequency Expert	1.244±0.213	0.303±0.027	1.870±0.460	0.419±0.068
Auxiliary-loss ablations				
w/o Reconstruction Loss	1.121±0.091	0.285±0.013	1.558±0.133	0.367±0.023
w/o SSL Loss	1.019±0.069	0.253±0.020	1.465±0.122	0.344±0.013

**Table A9:** Matched same-backbone comparison of no augmentation, single-degradation augmentation, unaligned full SDG, and the proposed patch-coordinate-aligned full SDG on the industrial SFD (L=720, H=16). Mean ± sample standard deviation over seeds 2023, 2024, and 2025. Best results in bold.

Variant	Clean		Spike		Noise		Shift		Stuck		Masking	
	MSE	MAE	MSE	MAE	MSE	MAE	MSE	MAE	MSE	MAE	MSE	MAE
w/o SDG	1.207±0.416	0.293±0.102	1.623±0.347	0.444±0.011	1.422±0.364	0.383±0.008	1.291±0.442	0.294±0.035	1.286±0.501	0.277±0.043	11.444±1.068	1.252±0.019
Simple Masking	1.029±0.061	0.254±0.007	2.772±0.299	0.705±0.053	1.475±0.115	0.408±0.042	3.130±0.052	1.102±0.006	0.997±0.063	0.253±0.007	3.287±0.198	0.748±0.021
Spike Only	1.738±0.500	0.392±0.104	1.810±0.466	0.410±0.086	2.519±0.319	0.519±0.064	2.474±0.457	0.513±0.075	2.059±0.270	0.450±0.061	4.050±0.530	0.674±0.076
Noise Only	1.441±0.402	0.353±0.085	2.580±0.994	0.510±0.126	1.807±0.456	0.420±0.079	1.970±0.454	0.441±0.073	1.530±0.392	0.363±0.077	3.767±0.629	0.627±0.092
Shift Only	1.050±0.003	0.270±0.008	1.925±0.080	0.420±0.026	1.867±0.242	0.410±0.028	1.284±0.015	0.331±0.007	1.222±0.045	0.301±0.003	2.136±0.115	0.456±0.013
Stuck Only	1.129±0.121	0.276±0.030	2.941±0.710	0.548±0.112	2.052±0.160	0.424±0.028	1.878±0.059	0.414±0.024	1.301±0.129	0.312±0.033	3.263±0.163	0.555±0.017
Unaligned Full SDG	1.185±0.022	0.305±0.005	1.653±0.234	0.407±0.039	1.807±0.132	0.424±0.022	1.447±0.036	0.372±0.008	1.282±0.031	0.322±0.003	1.532±0.055	0.379±0.002
Full SDG (Ours)	1.084±0.105	0.273±0.019	1.524±0.397	0.424±0.053	1.317±0.419	0.356±0.061	1.260±0.403	0.296±0.059	1.257±0.445	0.280±0.062	10.837±1.465	1.100±0.088

## Appendix D. Architectural Comparison with Existing Temporal-Frequency Forecasting Frameworks

This appendix provides a systematic architectural and functional comparison between SeTFiN and representative temporal-frequency forecasting models, including FEDformer, WaveRoRA, and AdaMamba. Table A10 summarizes their design differences across five core dimensions.

**Table A10:** Schematic comparison of SeTFiN with representative temporal-frequency forecasting approaches across core architectural dimensions.

Method	Frequency Representation	Temporal-Frequency Interaction	Sequence Backbone	Fusion Strategy	Degradation-Oriented Training
FEDformer [17]	DFT/DWT representation	Frequency modeling with seasonal-trend decomp.	Transformer	FEB + FEA + MOEDecomp	Not explicitly included
WaveRoRA [27]	Multiscale DWT coefficients	Joint multiscale wavelet modeling	Rotary Route Attention (RoRA)	WaveMixer + WaveSplitter	Not explicitly included
AdaMamba [22]	Adaptive frequency bases	Frequency analysis embedded in SSM updates	Mamba/Selective SSM	Adaptive frequency-gated update	Not explicitly included
SeTFiN (Ours)	CWT (Morlet wavelet)	Dual-pathway interaction with cross-domain rectification	Temporal & Frequency Mamba experts	CDFR + Adaptive fusion	Degradation-oriented dual-domain SDG

Note: FEB and FEA refer to Frequency Enhanced Block and Frequency Enhanced Attention, respectively. MOEDecomp denotes Mixture-of-Experts Decomposition. CDFR represents Cross-Domain Feature Rectification, and SDG stands for Self-Data Generation, a degradation-oriented data augmentation strategy used during training.

## Appendix E. Computational Efficiency and Complexity Analysis

Figure A1 compares the total test-set inference time of SeTFiN and representative baselines, while Table A11 reports complementary complexity indicators for SeTFiN.

**Table A11:** Complementary complexity profile of SeTFiN under representative forecasting settings.

Setting	Trainable Parameters	Latency (ms/batch)	Peak Allocated GPU Memory (MB)
ETTh1 (L=96, H=720, C=7)	1.223 M	0.837±0.124	39.28
SFD (L=720, H=16, C=17)	5.047 M	0.876±0.160	104.06

Note: Measurements used FP32 inference with a batch size of 1 on a single NVIDIA H200 NVL GPU. Latency is the mean ± sample standard deviation over 100 forward passes following 20 warm-up runs, and *C* denotes the number of input variables. The temporal expert, frequency expert, CDFR, and adaptive fusion contain 185,088, 349,056, 1072, and 33,410 trainable parameters, respectively. The fixed CWT kernels contain no trainable parameters but incur runtime computation and activation-memory costs. Total parameter counts differ because the prediction and auxiliary heads depend on the input and forecasting settings. FLOPs are omitted because the profiling tools used do not fully support the custom CWT and selective state-space operations.

## Appendix F. Detailed Formulation of SDG Operators and Implementation Settings

This appendix summarizes the SDG operators and implementation settings. SDG is applied to 40% of training batches. Within these batches, temporal degradation and masking positions are independently sampled with $r_{\deg}=0.15$ and $r_{\mathrm{mask}}=0.15$, respectively. The union of the selected degradation and masking indices is transferred to the CWT representation, where domain-specific coefficient perturbations are applied. Thus, temporal–frequency alignment denotes patch-index correspondence rather than exact transform consistency. The formulations and hyperparameters are summarized in Table A12 and Table A13.

**Table A12:** Mathematical formulations and hyperparameter configurations of the Self-Data Generation (SDG) operators.

Degradation Operator	Degradation/Perturbation Description	Mathematical Formulation	Hyperparameter/Distribution
Spike ($G^{\mathrm{spike}}$)	Transient amplitude increase	${\tilde{X}}_{T}\left[:,:,p,:\right]=\alpha X_{T}\left[:,:,p,:\right]$	α=3.0
Gaussian Noise ($G^{\mathrm{noise}}$)	Random measurement noise	${\tilde{X}}_{T}\left[:,:,p,:\right]=X_{T}\left[:,:,p,:\right]+\epsilon_{p}$	$\epsilon_{p}∼N\left(0,\sigma_{T}^{2}I\right),\;\sigma_{T}=1.5$
Signal Shift ($G^{\mathrm{shift}}$)	Bias or calibration shift	${\tilde{X}}_{T}\left[:,:,p,:\right]=X_{T}\left[:,:,p,:\right]+\delta_{p}1$	$\delta_{p}∼U\left(-2.0,2.0\right)$
Stuck Value ($G^{\mathrm{stuck}}$)	Constant sensor reading	${\tilde{X}}_{T}\left[:,:,p,t\right]=X_{T}\left[:,:,p,0\right],\;\forall t\in \left\{0,\ldots ,P-1\right\}$	Fixed to the first value in the selected patch
Random Masking ($G^{\mathrm{mask}}$)	Missing observation	${\tilde{X}}_{T}\left[:,:,p,:\right]=0$	$r_{\mathrm{mask}}=0.15$
Frequency-Domain Perturbation ($G_{F}$)	Position-aligned CWT perturbation	${\tilde{X}}_{F}\left[:,:,p,:,:\right]=\beta X_{F}\left[:,:,p,:,:\right]+\eta_{p}$	$\beta =0.1,\;\eta_{p}∼N\left(0,\sigma_{F}^{2}I\right),\;\sigma_{F}=0.5$

**Table A13:** Implementation settings used for SeTFiN.

Category	Parameter	Setting
Input/CWT	Patch length *P*	16
	Patch stride *S*	8
	Number of CWT scales	16
	Wavelet kernel length	15
	Wavelet parameterization	s=1,…,16, t∈[−3,3], $\psi_{s}\left(t\right)=e^{-t^{2}/2}\cos\left(5t/s\right)$
SDG	SDG application probability	0.40 of training batches (0.60 clean batches)
	Degradation ratio $r_{\deg}$	0.15
	Degradation duration	One patch at each selected position (P=16)
	Primary operator sampling	Spike/noise/shift/stuck: 0.25 each
	Spike	α=3.0
	Gaussian noise	$\sigma_{T}=1.5$
	Signal shift	δ∼U(−2.0,2.0)
	Stuck value	Fixed to the first value of the selected patch
	Random masking	$r_{\mathrm{mask}}=0.15$, sampled independently
	Frequency attenuation	β=0.1
	Frequency-domain noise	$\sigma_{F}=0.5$
	Temporal–frequency alignment	Same affected patch positions; domain-specific perturbations
Network	Model dimension $d_{\mathrm{model}}$	128
	Dropout	0.1
	Temporal/Frequency Mamba	$d_{\mathrm{state}}=16$, $d_{\mathrm{conv}}=4$, expand =2
	Front Network	16→32→16
	Rear Network	256→128→2
	Frequency Expert CNN	Two 3×3 convolutions, 16→128→128, padding =1, GELU
Training	Optimizer	AdamW
	Learning rate	$1\times 10^{-4}$
	Weight decay	$1\times 10^{-4}$
	Reconstruction-loss weight $\lambda_{1}$	0.05
	SSL-loss weight $\lambda_{2}$	0.005
	SSL temperature τ	0.2
	Positive-view CWT noise	$\sigma_{\mathrm{pos}}=0.03$
	Batch size	32
	Maximum training epochs	50
	Early-stopping patience	10

The reconstruction loss is the MSE between the reconstructed sequence and the original clean input window. For InfoNCE, the anchor is the projected representation of the current training view, and its positive is the same-sample representation constructed using clean temporal patches and CWT coefficients perturbed with $\sigma_{\mathrm{pos}}=0.03$. Representations of the other samples in the mini-batch serve as negatives.
